# Deficiency in transmitter release triggers homeostatic transcriptional changes that increase presynaptic excitability

**DOI:** 10.1073/pnas.2322714122

**Published:** 2025-07-29

**Authors:** Caroline A. Cypranowska, Maya Feldthouse, Yoon Gi Choi, Dariya Bakshinska, Rachel Li, Zachary L. Newman, Ehud Y. Isacoff

**Affiliations:** ^a^Department of Molecular and Cell Biology, University of California Berkeley, Berkeley, CA 94720; ^b^Helen Wills Neuroscience Institute, University of California Berkeley, Berkeley, CA 94720; ^c^Functional Genomics Laboratory, University of California Berkeley, Berkeley, CA 94720; ^d^Department of Neuroscience, University of California Berkeley, Berkeley, CA 94720; ^e^Weill-Neurohub East, University of California Berkeley, Berkeley, CA 94720; ^f^Molecular Biophysics and Integrated BioImaging Division, Lawrence Berkeley National Laboratory, Berkeley, CA 94720

**Keywords:** homeostasis, synapse, potassium channel, excitability, RNA-seq

## Abstract

Weakening of synaptic transmission at the *Drosophila* larval neuromuscular junction triggers two forms of homeostatic compensation, one that increases the probability of glutamate release per action potential (*P_r_*) and another that increases motoneuron (MN) activity. We investigated the molecular changes in MNs that underlie the increase in MN activity. RNA sequencing (RNA-seq) analysis on MNs whose glutamate release is weakened by knockdown of components of the MN transmitter release machinery reveals a reduction in expression of a group of genes that encode potassium channels and their positive modulators. These results identify a mechanism of compensation for weakened synaptic transmission by MNs, which engages a transcriptional program in those cells to increase firing and, thereby, ensure sufficient locomotory drive.

## Significance

The synaptic connections between neurons are dynamically regulated by plasticity mechanisms that strengthen or weaken particular synapses and by homeostatic mechanisms that adjust the overall system to ensure characteristic firing patterns needed to drive reliable behavior. In larval *Drosophila*, weakening of transmitter release by motor neurons is compensated for by increased motor neuron firing during locomotory bursts. To understand this compensation, we performed transcriptomics analysis on motor neurons whose transmitter release was reduced. We identify a set of genes, which encode K_v_ channels or modulators that boost K_v_ function, that are down-regulated when transmission is weakened. Increased motor neuron excitability due to reduced K_v_ function represents a cell-autonomous mechanism that can ensure sufficient motor output to drive locomotion.

To ensure reliable circuit function, the nervous system possesses homeostatic mechanisms that adjust neurotransmitter release to match postsynaptic sensitivity to transmitter, maintain a balance between excitatory and inhibitory inputs, restore total synaptic excitation to a transmission point following plasticity changes at a subset of synapses, while maintaining the newly differentiated synaptic weights, and regulate action potential (AP) firing to return to a firing set point following perturbation in order to ensure proper circuit output (1–3). In the *Drosophila* larval neuromuscular junction (NMJ), pharmacological or genetic reduction of the excitatory postsynaptic current (EPSC) through the GluRII glutamate-gated channel is compensated for by an increase in the amount of per AP glutamate release by type I MNs (4–7). Several distinct mechanisms appear to contribute to this presynaptic homeostatic plasticity (PHP), depending on whether the synaptic weakening is acute or chronic (4, 8), and in the case of acute PHP, the pharmacology of GluRII blockade (9). Remarkably, even when increased presynaptic release is insufficient to completely compensate for postsynaptic weakening, locomotor behavior is preserved (5, 10).

The *Drosophila* larval locomotory circuit has a second mechanism for compensation that appears to make up the difference. Mutation of the voltage-gated K^+^ channel ether-á-go-go (EAG) that blocks calmodulin-binding reduces AP-evoked glutamate release and increases type I MN excitability (11), suggesting a mechanism of firing compensation for synaptic weakening. This observation is complicated by the fact that the mutation is in a K^+^ channel that itself controls excitability. However, in support of this model, we recently observed that both postsynaptic, due to mutation of a subunit of the postsynaptic GluRII receptor, and presynaptic insults, due to knockdown of Cac, the voltage-gated Ca^2+^ channel that couples the AP to release, or of vesicle priming proteins Rbp or Unc-13, increase type I MN activity and preserves near normal locomotion (10). The molecular mechanism of this MN firing compensation is not known.

To probe the molecular mechanism of MN firing compensation, we examined the effect on gene expression of knockdown of the same presynaptic release components. We first confirmed that type I MN selective RNA interference (RNAi) knockdown of *Rbp*, *unc-13,* or *cac* severely reduces AP-evoked glutamate release. RNA-seq analysis revealed that these type I MN-selective knockdowns induce changes in expression of genes encoding ion channels, machinery proteins, and neuropeptides. Among these, a group of genes, which encode either voltage-gated K^+^ (K_v_) channels or their positive modulators and auxiliary subunits, stand out. Expression of these genes was reduced, consistent with increased excitability. We propose that attenuation of transmitter release from MNs triggers the coordinate reduction in expression of a homeostatic transcriptional module that normally suppresses AP firing to ensure constancy in circuit output and behavior.

## Results

**Fig. 1.**
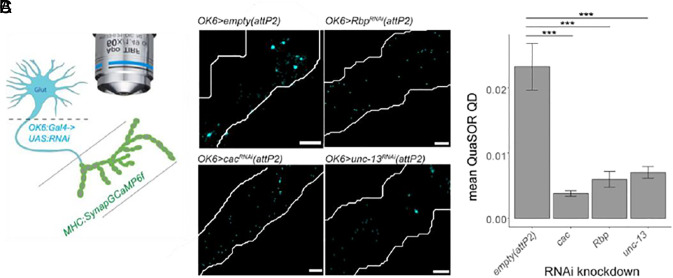
Weakened transmitter release from type Ib motor neurons by RNAi knockdown of components of the presynaptic transmitter release machinery. (*A*) Quantal of imaging synaptic transmission from Ib MNs by SynapGCaMP6f expressed in larval body wall muscle under MHC promoter (green) and OK6:Gal4 driving expression of UAS-RNAi in MNs (blue). (*B* and *C*) Cumulative transmission maps (*B*) and quantal density (average number of events per unit area of muscle) determined following QuaSOR analysis (*C*) in control [*attP2 (empty)*, n_Ib_ = 7] and UAS-RNAi for Rbp (n_Ib_ = 6), Unc13 (n_Ib_ = 5), or Cac (n_Ib_ = 5), each inserted at the *attP2* site. (Scale bar: 2 μm.) (*C*) Quantal density values are then mean ± SEM. (One-tail Student’s *t* test; ***P < 001).

Chronic reduction of glutamate release from type I MNs in the *Drosophila* larval NMJ elicits a compensatory increase in presynaptic firing to maintain circuit output (11). To understand the cell-autonomous molecular mechanisms in MNs that mediate this compensation, we performed RNA-seq on type I MNs whose glutamate release is compromised by knockdown of components of the presynaptic transmitter release machinery. To selectively perturb transmitter release in these neurons, we utilized OK6, a type I MN-specific Gal4 driver (12), to express RNAi constructs targeting three key release machinery components: i) RIM-binding protein (Rbp), which connects release sites to the scaffolding protein Bruchpilot (Brp), ii) Unc-13, a regulator of synaptic vesicle priming, and iii) Cacophany (Cac), the voltage-gated Ca^2+^ channel that couples the AP to transmitter release (13–17). Synaptic transmission events were measured with spinning disk confocal microscopy using SynapGCaMP6f, a fluorescent reporter which localizes to the postsynaptic density, and visualizes the Ca^2+^ component of the cation influx through the GluRII receptors that generate the excitatory postsynaptic current (Fig. 1*A* and *SI Appendix*, Fig. S1) (5, 18, 19). We applied our recently developed QuaSOR, Quantal Synaptic Optical Reconstruction, to enhance the spatial resolution of quantal transmission imaging (20). QuaSOR applies 2D Gaussian fits to transmission events to more precisely localize their origin, enabling discrimination of concurrent release events even in areas of densely packed synapses. As an optical measure of quantal content, we measured quantal density, the number of evoked transmission events per stimulus per unit area of postsynaptic membrane (5). In type Ib synapses, MN knockdowns of *Rbp*, *unc-13,* or *cac* reduced quantal density by ~70 to 85% (Fig. 1 *B* and *C*). To focus on perturbations that selectively weaken transmission, we focused on knockdown of *Rbp* and *unc-13*.

### Knockdown of Rbp and unc-13 in Type I MNs Induces Broad Changes in Gene Expression.

**Fig. 2.**
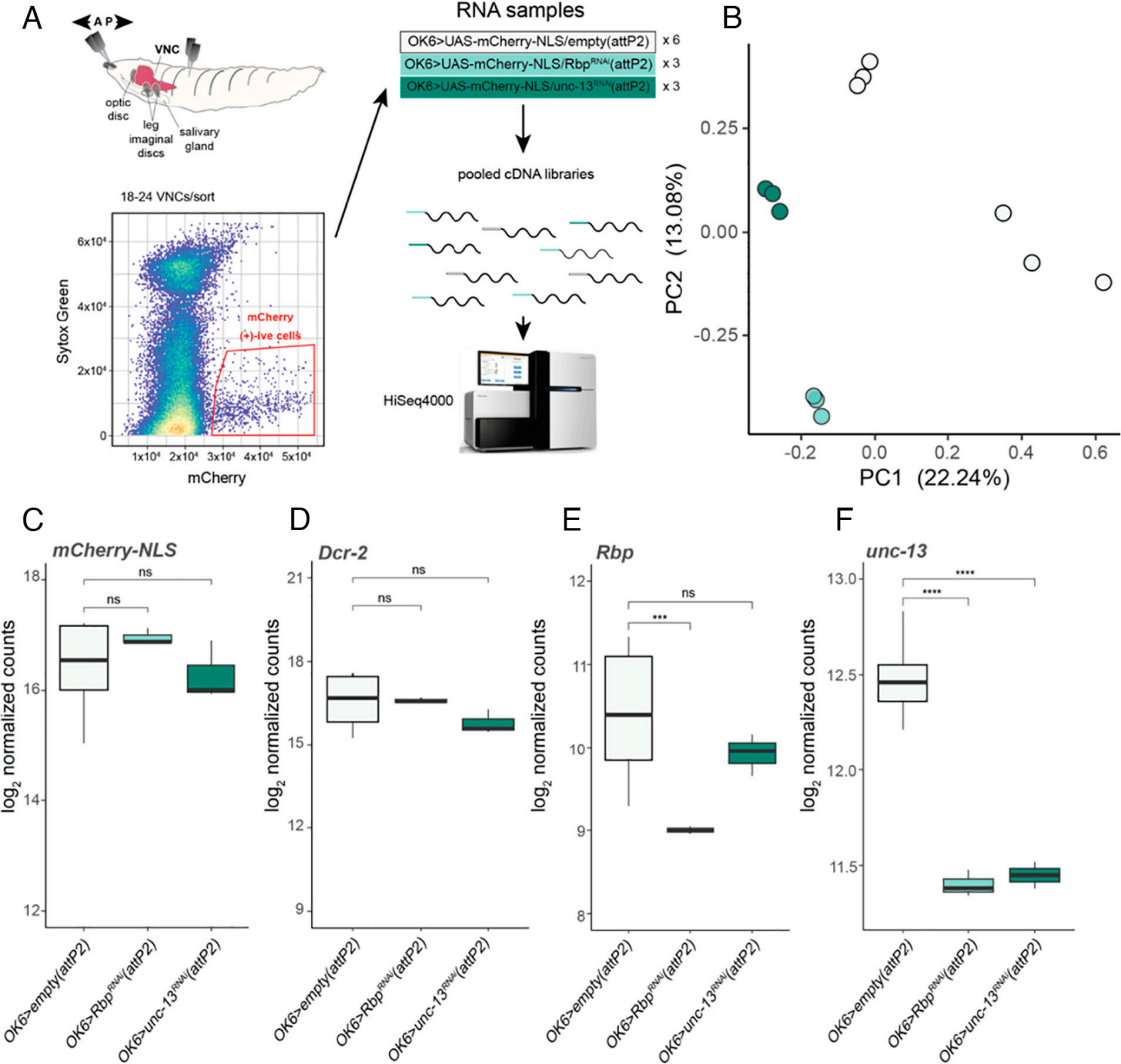
Knockdown *Rbp* or *unc-13* produces transcriptionally similar phenotypes. (*A*) Schematic for generating RNA-seq libraries from sorted motor neuron populations from control [*OK6>empty(attP2)*] and RNAi [*OK6>Rbp^RNAi^(attP2)*, *OK6>unc-13^RNAi^(attP2)*] animals. (*B*) PCA plot of RNA-seq samples. Library genotypes colored as depicted in (*A*). Axes are annotated by percent variance explained for each principal component. (*C*–*F*) Boxplots of log2 normalized pseudocount expression of (*C*) *mCherry-NLS*, (*D*) *Dcr-2*, (*E*) *Rbp*, and (*F*) *unc-13* (BH-adjusted Wald test *P*-values, ns *P* > 0.05, *P < 0.01, **P < 0.001, ***P < 0.0001, ****P < 1e-05).

To understand the molecular alterations that occur in type I MNs when synaptic transmission to muscle is profoundly compromised, we sequenced the transcriptomes of type I MNs, which carry the main excitatory drive to locomotory muscles in *Drosophila*. We compared controls to animals in which one of the components of the presynaptic release machinery is knocked down exclusively in the type I MNs. Type I MNs were isolated by expressing nuclear mCherry under the type I MN-specific *OK6-Gal4*, dissecting the ventral nerve cords, dissociating the cells, and capturing mCherry-expressing cells by fluorescence-activated cell sorting (FACS) (Fig. 2*A*). We extracted the RNA from this purified population of cells and prepared sequencing libraries from pooled sorted cell samples. This was done for control animals (*attP2*) and for animals that expressed the RNAi construct for either unc-13 [*OK6>unc-13*^RNAi^(*attP2*)] or Rbp [*OK6>Rbp^RNAi^(attP2)*]. Principal component analysis (PCA) of normalized libraries (Fig. 2*B*) shows that the samples of the same genotype cluster together and that samples from *OK6>Rbp^RNAi^(attP2)* and *OK6>unc-13^RNAi^(attP2*) are located closer to each other on the first principal component. This represents the axis of greatest variation between the datasets, indicating greater similarity between the transcriptomes of release machinery component knockdowns than between them and the control.

We assessed our normalization by testing for differential expression of *UAS* transgenes and dsRNA-mediated knockdown as ground truths. Samples from all three genotypes had similar expression of the *UAS-mCherry-NLS* reporter (Fig. 2 *C* and *D*). *OK6>Rbp^RNAi^(attP2)* samples had a 2.5-fold decrease in *Rbp* expression compared to the *OK6>empty(attP2)* controls, while the *OK6>unc-13^RNAi^(attP2)* samples had no difference in *Rbp* expression (Fig. 2*E*). Intriguingly, *unc-13* expression was reduced by about twofold in both the *OK6>unc-13^RNAi^(attP2)* and the *OK6>Rbp^RNAi^(attP2)* (Fig. 2*F*). The *OK6>unc-13^RNAi^(attP2)* samples had lower expression of the *UAS-Dcr-2* transgene (Fig. 2 *C* and *D*).

We were interested in the finding that loss of *Rbp* resulted in a concomitant decrease in *unc-13* at the transcriptional level. We therefore sought to quantify expression of Unc-13 at the AZ. To do this, we crossed the RNAi and empty *attP2* controls into an *OK6>Gal4;SynapGCaMP6f* line, which bears a GCaMP6f construct localized to the postsynaptic membrane (5). We labeled larval fillet preparations with an anti-GFP antibody to identify type Ib MN terminals, an anti-Brp antibody to identify AZs, and an antibody against Unc-13-A. Since the diameter of a Brp punctum in a wild-type Ib bouton is on the order of a few hundred nanometers (20, 21), we measured the fluorescence intensity by confocal imaging with Airyscan detection for improved spatial resolution (*Methods*). We found that Brp molecules were arranged in small, ring-like clusters as shown previously (20) (*SI Appendix*, Fig. S2 *A*–*C*) and that the Unc-13A intensity at the AZ was reduced by ~67% in the *Rbp* RNAi and 74% in the *unc-13* RNAi (*SI Appendix*, Fig. S2*D*).

### Knockdown of Rbp and unc-13 Increases Expression of Genes Encoding Neuropeptides and Their Secretion Machinery.

**Fig. 3.**
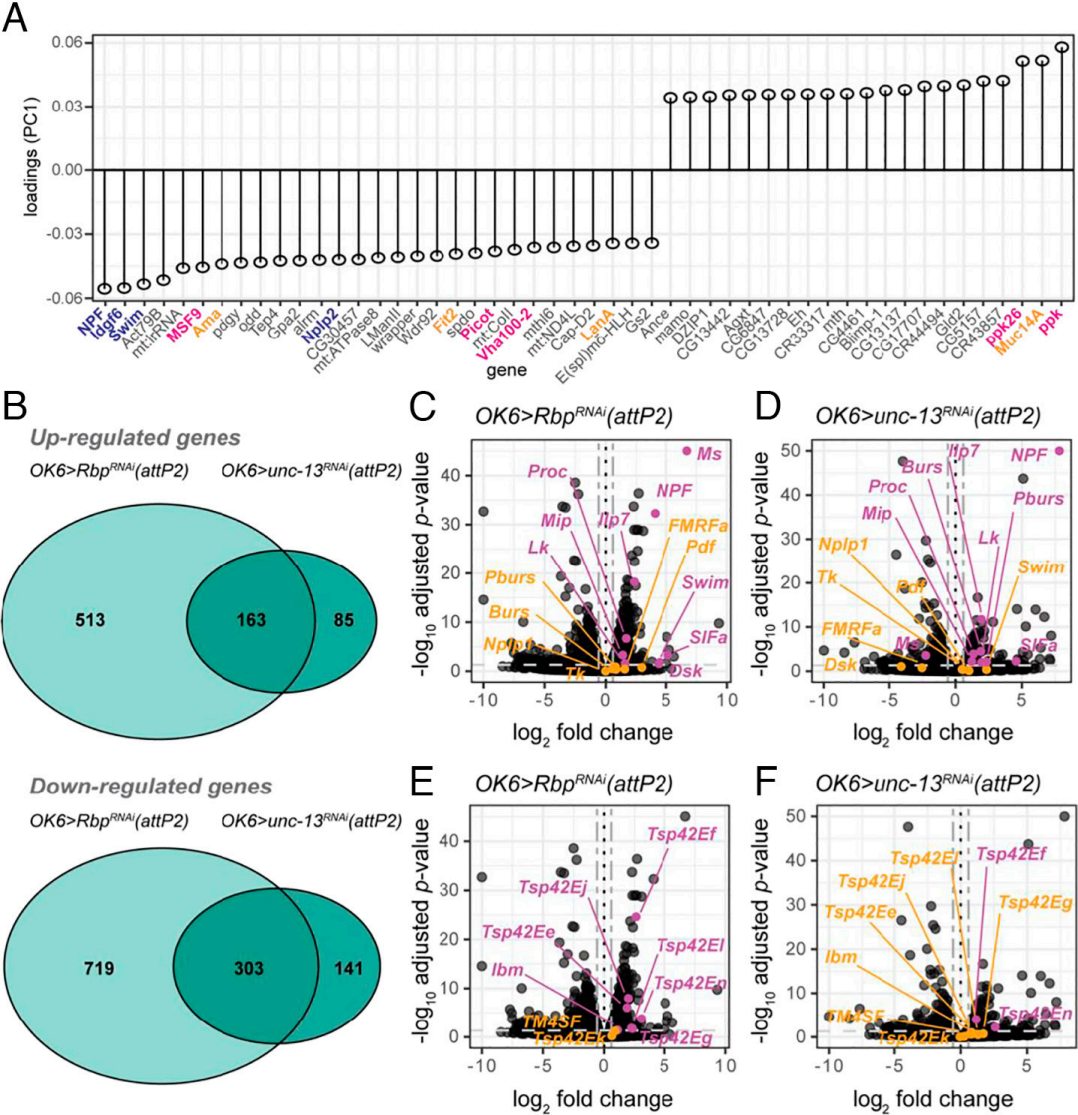
Rbp and unc-13 knockdowns yield overlapping changes in gene expression. (*A*) Weights of the top 50 genes contributing to PC1 ranked by magnitude (adjusted *P*-value < 0.05). Gene names in blue, magenta, and orange are neuropeptides and secreted proteins, ion channels and transporters, and cell–cell or cell–matrix adhesion proteins. (*B*) Venn diagram of differentially expressed genes (FDR = 0.05) by knockdown condition. (*C* and *D*) Volcano plots of *OK6>Rbp^RNAi^* (*C*) and *OK6>unc-13^RNAi^* (*D*) highlighting genes encoding neuropeptides and secreted proteins. (*E* and *F*) Volcano plots of *OK6>Rbp^RNAi^* (*E*) and *OK6>unc-13^RNAi^* (*F*) highlighting genes encoding exosome markers. Black dotted vertical lines in figures (*B*–*E*) demarcate a log_2_-fold-change of zero. The light gray single-dashed horizontal line demarcates an adjusted *P*-value of 0.05. The dark gray double-dashed vertical lines demarcate a fold-change of 0.5. Data points highlighted in magenta have an adjusted *P*-value < 0.05, while points highlighted in amber have a *P-*value ≥ 0.05.

To gain insight into the homeostatic alterations following synaptic perturbations, we asked what genes are differentially expressed in each RNAi compared to the *OK6>empty(attP2)* control. Since PC1 accounts for ~22% of variation in the transcriptomes for each genotype and represents the axis of greatest separation between the controls and RNAi knockdowns (Fig. 2*B*), we first examined the genes that contribute to the greatest loadings to this component (Fig. 3*A*). Examining the top 50 genes, we found the greatest magnitude of weights encoding neuropeptides (*NPF*, Neuropeptide F; *Nplp2*, Neuropeptide-like precursor 2), secreted proteinaceous ligands (*Swim*, Secreted Wnt-interacting molecule; *Idgf6*, Imaginal disc growth factor 6), ion channels and transporters (*ppk,* pickpocket; *ppk26*, pickpocket 26), and cellular adhesion proteins (*Fit2*, Fermitin 2; *LanA*, Laminin A; *Muc14A*, Mucin 14A). These results suggest that diverse changes in ion transport, excitability, and neuromodulatory signaling are common features of weakened excitatory transmitter release.

We next performed differential expression analysis to further characterize common features shared by each knockdown. *OK6>Rbp^RNAi^(attP2)* samples had more differentially expressed genes than did *OK6>unc-13^RNAi^(attP2)* samples (1,311 vs. 459) (Fig. 3*A*). The majority of differentially expressed genes in the *OK6>unc-13^RNAi^(attP2)* samples were also differentially expressed in the *OK6>Rbp^RNAi^(attP2)* samples (Fig. 3*B*), suggesting that *Rbp* has an upstream genetic interaction with *unc-13*. Among these, were genes encoding neuropeptides that were up-regulated in both *OK6>Rbp^RNAi^(attP2)* and *OK6>unc-13^RNAi^(attP2)* (Fig. 3 *C* and *D* and *SI Appendix*, Tables S2 and S3). The greatest increases in expression, which were shared between the two knockdowns, were for NPF and SIFa, whose central actions regulate a wide range of behaviors, including circuits controlling sleep, learning and memory, feeding, and metabolic homeostasis (22). Proctolin, which is found in a subset of type Ib MNs and their presynaptic nerve terminals, as well as in the type Is MNs projecting to dorsal muscle segments also increased in expression (23, 24), but to a lesser extent. We detected receptors in the type I MNs for two of these neuropeptides, NPFR and SIFaR, but not for proctolin (*SI Appendix*, Tables S4 and S5), which has been shown to act postsynaptically (24).

Since neuropeptides and other protein ligands are secreted from *Drosophila* motor neurons from large dense core vesicles or exosomes (25, 26), we also examined genes in the tetraspanin family, which are broadly involved in membrane organization and trafficking, but have specific roles in modulating glutamatergic transmission in the mammalian CNS (27). We found that several of the tetraspanins are up-regulated in *OK6>Rbp^RNAi^(attP2)* (Fig. 3*E* and *SI Appendix*, Table S2) and that two of those, *Tsp42En* and *Tsp42Ef*, are also up-regulated in *OK6>unc-13^RNAi^(attP2)* (Fig. 2*F*). We confirmed the increase in expression of *Tsp42Ef* with RT-PCR (*SI Appendix*, Fig. S3*A*).

### Knockdown of Rbp and unc-13 Decreases Expression of AZ Structural Components.

**Fig. 4.**
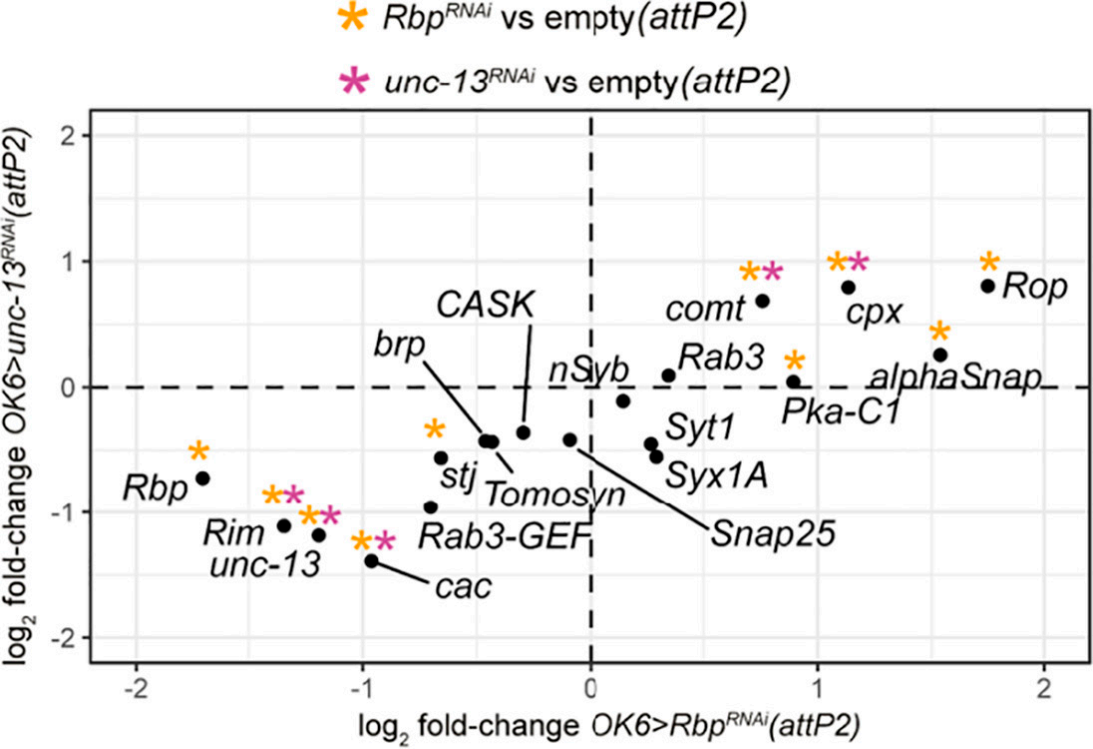
Knockdown *Rbp* or *unc-13* decreases the expression of AZ components. Log_2_ fold-change of *OK6>Rbp^RNAi^(attP2)* (horizontal axis) and *OK6>unc-13^RNAi^(attP2)* (vertical axis) highlighting genes comprising presynaptic transmitter release machinery. The log_2_ fold-change in expression for each genotype is compared to the *OK6>empty(attP2)* control. Black dotted lines demarcate a log_2_-fold-change of zero. [*P-value < 0.05. Asterisks in amber are DE in *OK6>Rbp^RNAi^(attP2)* and asterisks in plum are DE in *OK6>unc-13*^RNAi^*(attP2)*].

Our finding that the *Rbp* and *unc-13* knockdowns changes the expression of a genes involved in peptidergic signaling and trafficking led us to wonder whether altered expression of genes encoding other components of the transmitter release machinery could contribute to weakened transmitter release that we observed (Fig. 1 *B* and *C*). In both knockdowns, we observe decreased expression of *cac,* as well as of the cytomatrix AZ protein *Rim* (Fig. 4) (14–17). The *Rbp* knockdown has altered expression of additional genes encoding proteins that modulate the coupling between calcium entry and synaptic release. These genes include decreased expression of *unc-13*, as already mentioned, and of *straightjacket* (*stj*), the α2δ auxiliary subunit of the Cac channel (28), as well as increased expression of the fusion clamp *complexin* (*cpx*) (29), *Ras opposite* (*rop*) (30), the homolog of mouse *Unc18*) (31), *αSnap* (32), *comatose* (*comt*), the homolog of mouse *Nsf* (33), and the A-kinase *Pka-C1* (34, 35) (Fig. 4). Since Rbp, Unc-13, and RIM regulate coupling between synaptic vesicles and Cac, and Stj stabilizes Cac, these proteins collectively form the keystone of AZ organization (28, 36). Their decrease in expression, along with the increase in the Cpx fusion clamp, is consistent with the observed decrease in neurotransmitter release (37) (Fig. 1). These results were confirmed by RT-PCR for both *cac* and *Rim* (*SI Appendix*, Fig. S3*B*) and by immunohistological staining followed by Airyscan confocal imaging for Cac (*SI Appendix*, Fig. S4).

### Knockdown of Rbp and unc-13 Decreases Expression of Genes Encoding K_v_ Potassium Channels and Modulatory Subunits.

**Fig. 5.**
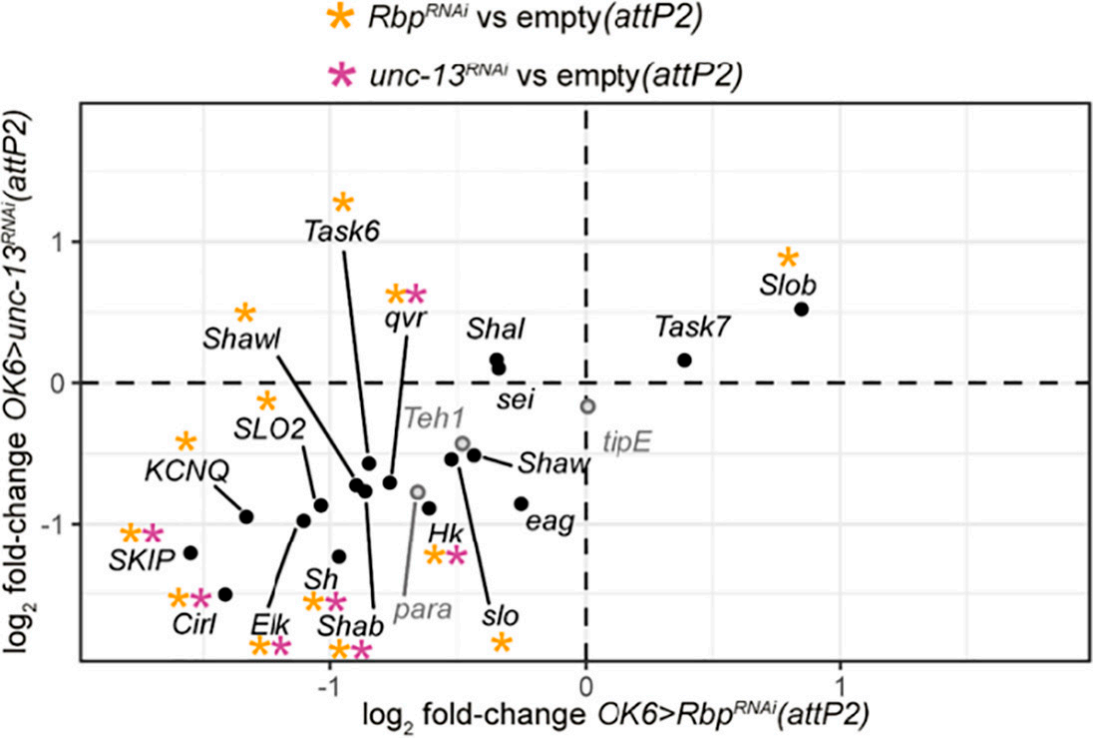
Knockdown *Rbp* or *unc-13* results in downregulation of suppressors of excitability. (*A*) log_2_ fold-change of *OK6>Rbp^RNAi^(attP2)* (horizontal axis) and *OK6>unc-13^RNAi^(attP2)* (vertical axis) highlighting genes encoding Kv channels and the aGPCR, Cirl (black) and Nav channels and associated proteins (gray). The log_2_ fold-change in expression for each genotype is compared to the *OK6>empty(attP2)* control. Black dotted lines demarcate a log_2_-fold-change of zero. [* indicates an adjusted *P-*value < 0.05. Asterisks in amber are DE in *OK6>Rbp^RNAi^(attP2)* and asterisks in plum are DE in *OK6>unc-13^RNAi^(attP2)*].

In an effort to understand how perturbations that reduce glutamate release by MNs increase their firing (4, 10, 11), we asked whether the *Rbp* and *unc-13* knockdowns also have altered expression of genes that regulate excitability. We found that in both the *Rbp* and *unc-13* knockdowns, MNs had reduced expression of three voltage-gated (K_v_) K^+^ channels, whose reduced function increases excitability: the A-type *Shaker* (*Sh*, K_v_1) channel (38, 39), the delayed-rectifier *Shab* (K_v_2) channel (39, 40), and the *ether a go-go* (*eag*) family member *Elk* channel (41) (Fig. 5 and *SI Appendix*, Tables S6 and S7). The KCNQ (K_v_7) (42) channel also had reduced expression, but only in the *Rbp* knockdown (Fig. 5 and *SI Appendix*, Table S6). There was no change in expression of several other K_v_ channels: *Shal* (K_v_3), *Shaw* (K_v_4), and *eag* and *SK*. We observed decreased expression of *Slob*, an auxiliary subunit of the *Slo* (BK) voltage and Ca^2+^ activated K^+^ channel (43, 44), but this only occurred in the *Rbp* knockdown (Fig. 5 and *SI Appendix*, Table S6). We next examined the expression of channel regulators and found that both the *unc-13* and *Rbp* knockdowns had decreased expression of three positive regulators of K_v_ channels: *Hyperkinetic* (*Hk*) (45–47), *quiver* (*qvr/*SSS) (48, 49), and *SKIP* (50) (Fig. 5 and *SI Appendix*, Tables S5 and S6). Of these, loss of *Hk* and *qvr* has each been experimentally shown to reduce K+ current and increase neuronal excitability (47, 49). We confirmed the decreased expression of *SKIP*, *Shab*, *qvr,* and *Hk* by RT-PCR, using *eag* as a control (*SI Appendix*, Fig. S3*C*).

In addition to these changes in genes encoding K+ channels and their modulatory subunits, both the *unc-13* and *Rbp* knockdowns had a reduction in the expression of *Cirl* (Ca^2+^ Independent Receptor for Latrotoxin) (Fig. 5 and *SI Appendix*, Table S6), a Gi-coupled adhesion GPCR whose loss may contribute to increased firing (51).

In contrast to the decreased expression of K^+^ channels and their modulators in the *Rbp* and *unc-13* knockdowns, there was no change in expression of the voltage-gated sodium channel (*para*) (52), or of two *para* β subunits, *tipE* (53, 54) and *Teh1* (55), or of a *para* genetic regulator, *pasilla* (*ps*) (56, 57) (Fig. 5 and *SI Appendix*, Tables S5 and S6). This implies that, rather than a generalized decrease in channel expression, K_v_ channels and modulators are specifically regulated to compensate for synaptic weakening.

Because knockdown of *Rbp* also reduced the expression of *unc-13*, we sought to examine an additional synaptic release machinery component. Since RNAi knockdown of *cac* in type I MNs also reduces quantal density of transmission (Fig. 1*C*), we performed RNA-seq on *cac* knockdown in type I MNs as for *Rbp* and *unc-13* knockdowns (*SI Appendix*, Fig. S5 *A*–*D*). We found decreased expression of three K^+^ channels (*Shaker*, *KCNQ,* and *SK*) and four K^+^ channel positive modulators (*Hk*, *SKIP*, *Slob,* and *Cirl*), without a significant change in expression of *unc-13* (*SI Appendix*, Fig. S5 *E* and *F* and Table S13). As we observed with the *Rbp* and *unc-13* knockdowns, we found no change in expression.

We then wanted to look upstream in the motor circuit, so we also asked whether *Rbp* or *unc-13* knockdown results in altered expression of ionotropic neurotransmitter receptors that could affect the response to synaptic input from premotor interneurons. *Drosophila* interneurons fall into three classes—cholinergic (excitatory), GABAergic (inhibitory), or glutamatergic (primarily inhibitory via *GluCl*α glutamate-activated Cl- channels) (58–63). We examined the expression genes encoding synaptic receptors to glutamate, acetylcholine, or GABA, which represent the major transmitters that premotor inputs release onto MNs (64). We found no change in expression for the majority of genes encoding transmitter receptors (*SI Appendix*, Tables S10 and S11). However, in the *unc-13*, *Rbp*, and *cac* knockdowns, we observed decreased expression of both the excitatory nicotinic acetylcholine receptor *nAChRalpha6* and of the inhibitory GABA-A receptor subunit *Rdl* (*SI Appendix*, Tables S10, S11, and S14). Furthermore, expression of the excitatory AMPA-type ionotropic glutamate receptor *GluRIB* and the inhibitory *GluCl*α was reduced in the knockdowns of *unc-13* and *Rbp* but not *cac*. However, it should be pointed out that, in addition to its effect on *Shaker* current, *qvr* inhibits acetylcholine receptor activity (49), so that reduced *qvr* expression increases cholinergic synaptic excitation. However, the change in expression of *qvr* was observed in the knockdown of *unc-13* and *Rbp* but not *cac*. The overall anticipated outcome of these changes in expression of transmitter receptors on synaptic excitation is mixed, preventing a clear conclusion about the overall effect on MN firing. Overall the consistent aspect of our observations is that chronic presynaptic disruption of transmitter release from motor neurons leads to an orchestrated decrease in expression K^+^ channels and their positive modulators.

## Discussion

### Homeostatic Coupling of AP Firing to Synaptic Transmission.

Following changes to neural activity or synaptic transmission that occur during development or as a result of synaptic plasticity, neuromodulation, or pathological perturbation, homeostatic mechanisms adjust neural properties to return AP firing and synaptic transmission to set points that ensure proper circuit output (1–3). At the model glutamatergic synapse of the *Drosophila* larval NMJ, retrograde signaling from postsynaptic muscles to presynaptic type I MNs triggers PHP, which has been characterized as the increase in AP-evoked glutamate release that compensates for a decrease in the amplitude of the excitatory postsynaptic potential due to either a null mutation of the GluRIIA subunit of the GluRII ionotropic glutamate receptor or to pharmacological block of GluRII (4, 7). However, the chronic, genetic form of this synaptic disruption boosts glutamate release only in one of the two convergent type I glutamatergic MN inputs to the body wall muscle, the type Ib MN, whereas the acute, pharmacological form only occurs in type Is MNs (5, 9). In other words, this PHP mechanism only partially compensates for weakened synaptic transmission (20). Despite this, locomotory behavior of the *GluRIIA^−/−^* animals remains near normal (10). Moreover, animals also locomote near normally when they have presynaptic knockdown of any one of three components of the glutamate release machinery—*unc-13, Rbp,* or *cac*—which severely reduces AP-evoked release, without detectable postsynaptic compensation (10). These presynaptically and postsynaptically weakened animals show a common change in MN activity pattern: They increase the duration of activity bursts that drive the locomotory peristaltic waves of contraction. This indicates that there is a second mechanism of homeostatic regulation that compensates for weakened per AP synaptic transmission from MN to muscle in which the MNs fire more APs, as suggested earlier (10).

We probed the mechanism of MN firing homeostasis by characterizing the transcriptional consequences of impaired glutamate release due to knockdown of the same presynaptic components of the transmitter release machinery, *unc-13* and *Rbp*. We find that knockdown of *unc-13* or *Rbp* changes the expression of an overlapping set of genes, and knockdown of *Rbp* itself reduces *unc-13* expression, suggesting that there is a hierarchical relationship between the two genes. It follows that both the overlapping changes in gene expression and much of the presynaptic weakening are due to the reduction in *unc-13.* To investigate this further, we examined the transcriptome of type I MNs expressing an RNAi construct against *cac*, which also reduces the quantal density of synaptic transmission (Fig. 1*C*). Reduced *cac* expression results in decreased *Rbp* expression, but *unc-13* expression is not changed significantly (*SI Appendix*, Fig. S5). While paradoxical at face value, decreased expression of one release machinery component resulting in the loss of other proteins in the complex has been observed before. For example, *Brp*-null mutant NMJs have decreased Cac at the AZ (65) and Rab3 mutants have fewer AZs with altered distribution of AZ components (66). These results suggest that AZ proteins assemble into functional structures in a coordinated fashion and that loss of one AZ component is not replaced by gain of another.

### Changes in Expression of Genes Encoding Channels and Modulators That Regulate Excitability.

Rather than increased expression of genes encoding presynaptic release machinery, we observe in all knockdowns the decreased the expression of a group of genes that encode pore forming α-subunits of K_v_ channels or modulatory proteins that enhance their function. These include the inactivating (I_A_) Shaker (K_v_1) channel, the delayed rectifier Shab (K_v_2) channel, and the Eag-like Elk (K_v_12) channel. Additionally, in the *Rbp* knockdown, we also see reduction in expression of the voltage- and Ca^2+^-gated Slo (BK) and voltage-gated Shawl (K_v_4) and KCNQ (K_v_7) K^+^ channels. Reduced K^+^ channel expression is known to increase the excitability of type I MNs (39, 41, 67). More important still, Shaker and Shab mutants have each been shown not only to increase AP firing, but to increase the duration of MN locomotory bursts (39), exactly as observed in intact larvae during restrained locomotion with knockdown of *unc-13* and *Rbp* (10). Indeed, direct knockdown of either Shaker or Shab in type I MNs has the same effect as weakening of synaptic transmission of increasing the duration of type I MNs locomotory transmission bursts (10). In other words, reduced expression of these K_v_ channels can account for the compensatory alteration of firing patterns that support locomotor behavior.

Both the *unc-13* and *Rbp* knockdowns also reduced expression of three genes encoding K^+^ channel modulatory subunits that boost K_v_ channel function: the Shaker interacting proteins *Hk* and *qvr/*SSS, as well as the Shal regulator SKIP. *Hk* is a β subunit of the Shaker channel, which boosts channel current amplitude, accelerates current rise and shifts the voltage dependence of activation to more negative voltage (45, 46). Elimination of *Hk* resembles reduction of *Shaker* expression and leads to hyperexcitability (47). *Qvr/*SSS is an Ly6/GPI-anchored protein, whose loss slows Shaker channel current rise and increases cumulative inactivation, resulting in a decreased Shaker current amplitude and increased excitability (48, 49). *SKIP* slows the inactivation of Shal channels and its elimination results in fast inactivation (i.e., reduction of K+ current) and greater excitability (50). Expression was also reduced in *Slob*, an auxiliary subunit of the *Slo* (BK) voltage and Ca^2+^ activated K^+^ channel (43), but this only occurred in the *Rbp* knockdown (Fig. 5 and *SI Appendix*, Table S6). Deletion or knockdown of *Slob* increases AP firing in Drosophila type I MNs (44) and enhances glutamate release (68). In addition, both the *unc-13* and *Rbp* knockdowns had a reduction in the expression of *Cirl* (Ca^2+^ Independent Receptor for Latrotoxin) (Fig. 5 and *SI Appendix*, Table S6). Cirl is a Gi-coupled adhesion GPCR whose activity reduces cAMP levels and protein kinase A activity in cells (69). Loss of function of Cirl may also contribute to increased firing (51). Thus, reduced expression of these K_v_ channel modulators and Cirl may further contribute to the compensatory alteration of firing patterns that rescue locomotor behavior.

It should be noted that we did not observe complete loss of expression of these genes, only reductions by 30 to 65% compared to controls. However, increased excitability is also seen in hypomorphs and heterozygotes with one wild-type copy of the gene (38). This suggests that reduction by about half, as we observe here in the *unc-13* and *Rbp* knockdowns, is sufficient to increase firing, especially when there is a simultaneous decrease of this magnitude in multiple genes.

Taken together, weakening of glutamate release by type I MNs coordinately reduces expression of genes encoding K_v_ channels and their function-boosting associated proteins. We propose that cosuppression of this gene module functions as a mechanism for increasing the number of APs in locomotory bursts to compensate for reduced glutamate release per AP. Intriguingly, three of these channels—Shaker, Shab, and Slo—have already been found to coordinately increase in expression in response to elimination of the Shal (K_v_3) K^+^ channel (70), supporting the notion that they operate as a coregulated transcriptional module, though K^+^ channel compensatory mechanisms are complex.

## Conclusion

Weakening of glutamatergic transmission from *Drosophila* MNs to muscle engages two multiplicative mechanisms of homeostasis: PHP, which boosts the amount of transmitter release per AP (4), and increased duration of MN bursts, which can further boost release by increasing the number of APs that drive the contraction wave of locomotion (10, 11). We find here that molecular disruptions that reduce AP-evoked glutamate release, resulting in compensatory firing homeostasis, drive a coordinated reduction in expression of a group of K_v_ channels and modulatory proteins that augment K_v_ channel function. These transcriptional changes provide a molecular mechanism for the compensatory increase in MN locomotory burst duration in behaving animals that preserves locomotion in the face of synaptic perturbations (10).

## Methods

### Fly Stocks.

As described recently (71), the *UAS-Rbp^RNAi^* line (TRiP.JF02471, BDSC #29331), *UAS-unc-13^RNAi^* line (TRiP.JF02440, BDSC #29548), *UAS-cac^RNAi^* line (TRiP.JF02572, BDSC #27244), and the empty *attP2* acceptor line (BDSC #36303) were obtained from the Bloomington Drosophila Stock Center as donated by the Transgenic RNAi Project. *UAS-dsRNA* constructs were driven in type I MNs with *OK6-Gal4* (12, 72). *UAS-Dcr2* (BDSC #24646) was used to improve the efficacy of RNA interference (73). Fruit fly stocks bearing a synaptically localized GCaMP6f (*SynapGCaMP*) under the control of the myosin heavy-chain (*MHC*) promoter were used for reporting postsynaptic activity in the muscle (5) in electrophysiology experiments. For RNA-seq experiments, fruit fly stocks bearing transgenes encoding nuclear mCherry (*UAS-mCherry-NLS*, BDSC #38424, ref. 74), along with Dcr2 and dsRNA constructs, in type I MNs were used for the purpose of FACS.

Also, as described recently (71), larvae were raised to the 3rd instar stage on cornmeal and molasses media in an incubator at 25 °C. Female parents bearing the *OK6-Gal4* driver and *UAS-Dcr2* transgenes were crossed with male parents bearing either the *attP2* empty vector or RNAi construct to obtain control and knockdown progeny, respectively. 3rd instar larvae with the following genotypes were used for RNA-seq:

*UAS-Dcr2;OK6-Gal4/+;UAS-mCherry-NLS/(attP2)* (*abbr. OK6>empty(attP2)*)

*UAS-Dcr2;OK6-Gal4/+;UAS-mCherry-NLS/UAS-Rbp^RNAi^(attP2)* (abbr. *OK6>Rbp^RNAi^(attP2)*)

*UAS-Dcr2;OK6-Gal4/+;UAS-mCherry-NLS/UAS-unc-13^RNAi^(attP2)* (abbr. *OK6>unc-13^RNAi^(attP2)*)

*UAS-Dcr2;OK6-Gal4/+;UAS-mCherry-NLS/UAS-cac^RNAi^(attP2)* (abbr. *OK6>cac^RNAi^(attP2)*)

3rd instar larvae of the following genotypes were used for immunohistochemistry and optical quantal analysis experiments:

*UAS-Dcr2;OK6-Gal4/+;MHC-SynapGCaMP6f/(attP2)*

*UAS-Dcr2;OK6-Gal4/+;MHC-SynapGCaMP6f /UAS-Rbp^RNAi^(attP2)*

*UAS-Dcr2;OK6-Gal4/+;MHC-SynapGCaMP6f /UAS-unc-13^RNAi^(attP2)*

*UAS-Dcr2;OK6-Gal4/+;MHC-SynapGCaMP6f /UAS-cac-13^RNAi^(attP2)*

### SynapGCaMP6f Optical Quantal Imaging.

#### Optical quantal imaging.

Optical quantal imaging was performed similarly to our previous reports (19, 20). As described recently (71), third instar larvae were dissected on PDMS (Sylgard 184, Dow Corning, Auburn, MI) pads in ice-cold HL3 solution containing, in mM: 70 NaCl, 5 KCl, 0.45 CaCl2, 20 MgCl2, 10 NaHCO3, 5 trehalose, 115 sucrose, 5 HEPES, and with pH adjusted to 7.2. Following removal of the brain (VNC), larval fillets were washed and imaged in room temperature HL3 containing 1.5 mM Ca^2+^ and 25mM Mg^2+^. Fluorescence images were acquired at room temperature with a Vivo Spinning Disk Confocal microscope (3i Intelligent Imaging Innovations, Denver, CO), using a 63 × 1.0NA water immersion objective (Zeiss), 1.2× optical adapter, LaserStack 488 nm (50 mW) laser, CSU-X1 A1 spinning disk (Yokogawa Tokyo, Japan), standard GFP filter, and EMCCD camera (Photometrics Evolve512, Tucson, AZ). All live SynapGCaMP6f imaging recordings were done on ventral longitudinal abdominal muscle 4 at segments A3-A5 of third instar larvae. All imaging was performed using 50 ms exposures (20 fps) of the full camera sensor (512 × 512 px). Nerve stimulation was performed with a suction electrode attached to a Stimulus Isolation Unit (SIU, ISO-Flex, A.M.P.I. Jerusalem, Israel), with 100 μs stimulus duration. The intensity of the stimulus was adjusted to recruit both Ib and Is axons (verified through imaging) and kept constant throughout the imaging session. Nerve stimulation and imaging were synchronized using custom-written Matlab scripts (Matlab Version 2015b, MathWorks, Inc., Natick, MA) in order to control the SIU and trigger imaging episodes with SlideBook (v6.0.16, 3i Intelligent Imaging Innovations). For comparability between experiments, recordings were done on only one NMJ (Ib–Is pair) per larva, and recordings were performed within 30 min from the start of the dissection, ensuring the animals’ health. As in our previous report (20), we alternated which segments (A3-A5) we imaged from since no significant difference was found between segments.

#### Functional registration and bleach correction.

As described recently (71), the initial quantal image analysis was performed using custom-written MATLAB protocols, the same as in our previous work (20). Individual stimulus episodes were excluded due to out-of-focus NMJs, moving NMJs, or failed axon recruitment. Otherwise, all movies were filtered (Gaussian low-pass filter), to reduce high-frequency noise. Image analysis areas were then separated into Ib and Is NMJ regions. All imaging data were registered using a multistage approach, during which all images were registered to a common reference image (usually the first frame of the first image).

Following area selection and reference image selection, NMJs were tracked relative to this reference image using a rigid subpixel registration method to remove any large movements within the NMJ imaging area (19, 20, 35). As in our previous work (20), we corrected for local bouton movements using a custom diffeomorphic implementation of a demons algorirthm (36) Once motion corrected, movies were bleach corrected using a fit for a double exponential bleach correction curve to the mean baseline pre- and poststimulus fluorescence data for each trial separately. Following bleach correction, both ΔF and ΔF/F movies were generated using the first image as the baseline fluorescence (F0) image for the episodic data.

#### QuaSOR.

After event detection, isolation, and verification, we then proceeded to analyze the 2D response profile of each event’s maximum ΔF/F spatial profile using the custom QuaSOR algorithm as in our previous work (20). As described recently (71), we first looked at all the identified responses from our quantal detection and isolated small ROIs containing individual or small groups of partially overlapping response fields that corresponded to individual or small groups of events. These smaller ROIs were then subjected to independent 2D Gaussian mixture model fitting of the isolated ΔF/F spatial profiles. Gaussian mixture model fitting for all response ROIs, all event functions were then remapped onto a common coordinate space and merged to define a single set of 2D Gaussian functions for each quantal response. The peak positions of each 2D Gaussian component were used to define event locations in a 21.2 nm × 21.2 nm pixel coordinate space. For visualization purposes, maps were generated by applying a normalized 2D Gaussian filter to each event coordinate prior to adding each event to the overall image. In this way each pixel contains an approximation of the event density at that location. Local QuaSOR synapse alignments were performed by identifying maximum evoked coordinate density positions for synaptic ROIs within a 350 nm radius. Maintaining their relative organization to nearby events, these QuaSOR event coordinates were averaged together with other synapses to generate a mean density image for synapse groupings.

### CNS Dissection.

As described recently (71), prior to dissection, 3rd instar larvae were screened for the presence of nuclear mCherry in the VNC with either a ZEISS Axio Zoom v16 FL epifluorescence microscope or a stereo dissection microscope. For bulk RNA-seq experiments, larvae were rinsed twice in 1× PBS (Gibco), once in 70% ethanol, and again in 1× PBS to sterilize the surface of the animals. Larvae were dissected on a Sylgard pad in a droplet of Schneider’s complete media (Gibco) supplemented with 10% FBS (Corning) and 1% PenStrep (Gibco). After removing extraneous tissues, the brain and VNC were transferred to a low-binding 1.5 mL tube containing Schneider’s complete media on ice until dissociation. Dissected tissues were kept on ice prior to dissociation for no longer than 60 min to ensure the viability of the cells during FACS.

### CNS Dissociation.

As described recently (71), tissue samples were washed three times with 500 μL Schneider’s media containing 1.5 mM EDTA and 1.5 mM L-Cysteine to remove media containing FBS. Fresh dissociation solution was prepared for each experiment from Schneider’s media with the following additives: 1.5 mM EDTA, 1.5 mM L-Cysteine, papain (Worthington), and 1.25 mg/mL collagenase type-I (MilliporeSigma). Tissue samples were transferred into the dissociation solution and incubated at 25 °C on a nutator for 5 min. Samples were triturated 30 times with a P200 pipette tip. This process of incubation and trituration was repeated before a final step in which tissue was sheared 7 times with a 27G1/2 needle.

The digestion reaction was quenched with the addition of 500 μL Schneider’s complete media with 10% FBS and 1% PenStrep. Dissociated cells were passed through a 40 μm cell strainer (Falcon) and transferred to a low-binding 1.5 mL tube. The processed sample was optionally centrifuged at 0.7 × g for 7 min, after which the supernatant was replaced with 1 mL of Schneider’s complete media containing 5% FBS and 1% PenStrep.

### Fluorescence-Activated Cell Sorting.

As described recently (71), processed cell samples were stained with 0.3 μM Sytox Green (Molecular Probes) to assay cell viability prior to sorting. Cells were sorted on a BD Influx Cell Sorter with 140 μm tubing at pressures < 12 PSI. The sorted population was chosen for high expression of nuclear mCherry (PE-Texas Red) and low Sytox Green fluorescence (GFP). Plots of FACS data were created using the flowCore (ver 1.52.1) (75) and ggcyto packages (ver 1.14.0) (76).

### Low-Input RNA-seq Library Preparation and Sequencing.

As described recently (71), cells were sorted into RLT lysis buffer (RNeasy Micro, QIAGEN) supplemented with 3.5 mM BME. Cells were lysed by vortexing for 30 s at room temperature. RNA extraction was performed according to the RNEasy Micro kit protocol, omitting the DNase digestion step. Extracted RNA was checked for quality by an Agilent 2100 Expert Bioanalyzer with a Eukaryote Total RNA Pico chip.

Sequencing libraries were prepared with either the SMART-Seq2 protocol (*SI Appendix*, Table S1) or the Takara SMART-Seq v4 Ultra-low-input RNA kit (*SI Appendix*, Table S12). RNA prepared with the SMART-Seq2 protocol (*SI Appendix*, Table S1) was annealed to an oligo-dT primer (5′-AAGCAGTGGTATCAACGCAGAGTACT_30_VN-3′, IDT) in a buffer containing 2.5 mM dNTPs and recombinant RNase Inhibitor (Takara). First-strand synthesis was performed with the SuperScriptII kit using a custom template-switching oligo (5′-/5Me-isodC//iisodG//iMe-isodC/AAGCAGTGGTATCAACGCAGAGTACATrGrGrG-3′, IDT) with 5′ modifications to avoid the creation of concatemers in low-input samples (77). SMART-Seq2 library cDNAs were digested with lambda exonuclease (New England Biolabs) digestion prior to amplification as described previously (78), whereas SMART-Seq v4 library cDNAs were fragmented with a S220 Focused-Ultrasonicator (Covaris).

SMART-Seq2 libraries were constructed using the Nextera XT kit (Illumina) and size-selected with AMPure XP beads, followed by size selection with Pippin Prep (SAGE Biosciences), followed by 100-nt paired-end sequencing on an Illumina Hi-Seq 4000 instrument. SMART-Seq v4 libraries were prepared with the KAPA HyperPrep kit for DNA (KK8504). Truncated universal stub adapters were used for ligation, and indexed primers were used during PCR amplication to enrich the libraries for adapter-ligated fragments. SMART-Seq v4 libraries were sequenced on an Illumina NovaSeq X to generate 150-nt paired-end reads.

### RNA-seq Alignment and Preprocessing.

As described recently (71), demultiplexed .fastq files were first analyzed with FastQC (ver 0.11.7) (79) to check for quality and trimmed with Trimmomatic (ver 0.38) (80). Trimmed reads were aligned to a custom reference genome built from the Berkeley Drosophila Genome Project (dm6, release 98) genome assembly and the pUAST-mCherry-NLS vector sequence for the detection of the mCherry messenger RNA (mRNA) reads. Samples bearing double-stranded RNA (dsRNA) constructs were mapped to custom genomes containing the aforementioned transgenes and the sequence of the dsRNA construct. Mapping was performed with HISAT2 (ver 2.1.0) (81), and the counts matrix was generated with the featureCounts function from the Subread package (ver 1.6.4) (82).

### Gene Filtering, Normalization, and Differential Expression Analysis.

As described recently (71), biological analysis was performed in R after using a custom script to import the featureCounts output into a SummarizedExperiment object (ver 1.16.1) (83). Genes with more than 5 counts in at least 3 libraries were considered detectable. Genes that did not meet this threshold were removed from downstream analysis. Libraries were normalized by calculating a size factor for each sample from the median ratio of gene expression relative to the geometric mean for each gene as implemented in DESeq2 (ver 1.26.0) (84). Identification of differentially expressed (DE) genes between each knockdown condition was performed with DESeq2. Visualization of DE analysis was performed with custom scripts built upon ggplot2 (ver 3.2.1) (85).

### qRT-PCR.

FACS and RNA purification sorted cells for qRT-PCR experiments were prepared as described for the low-input RNA-seq experiments. cDNA was reverse transcribed from purified RNA with the SuperScript III First-strand Synthesis System (Invitrogen) using 5 μM oligo(dT)_20_ as a primer to improve selectivity for mRNA in the samples. qPCR was performed with the Power SYBR Green PCR Master Mix (Applied Biosystems) on a BioRad CFX96 Touch Real-Time PCR Detection System. Each reaction was performed in triplicate along with no-template controls.

### Primer Design.

Primers for the qPCR experiments were designed using FlyPrimerBank (86, 87), with the exception of the primer pair targeting EF1a which was previously published by Ponton et al (88). Primers were selected by their annealing temperature, favoring primer pairs that span regions of the target gene present in all known isoforms and span exonic regions to reduce the likelihood of genomic DNA amplification. Sequences for each primer used in this study are in *SI Appendix*, Table S15.

#### Quantification of expression.

Expression for each gene of interest was determined with the relative quantification method with correction for differences in primer efficiency (89). aTub84B and EF1a were chosen as reference genes for relative quantification of gene expression across controls and knockdowns.

### Immunohistochemistry.

Larval fillets were prepared as described previously (20). As described recently (71), larvae were fixed at room temperature in Bouin’s fixative (Ricca Chemical Company, Arlington, TX) for 5 min and rinsed with ice-cold PBS with 0.1% Triton X100 (PBT) to remove trace fixative. Larval fillets were blocked in PBS with 0.1% Triton X100, 2.5% normal goat serum, and 0.02% sodium azide (PBN) for 30 min. All antibody incubations were performed in PBN. For primary antibody staining, mouse anti-Brp (nc82; Developmental Studies Hybridoma Bank, Iowa City, IA) was used at 1:150, while rabbit anti-unc-13A (16) and rabbit anti-Cac (90) were used at 1:1,500. All primary antibody incubations were performed overnight at 4ºC. Samples were washed three times for 15 to 20 min in PBT at room temperature prior to blocking and secondary antibody labeling. Alexa Fluor Plus 555 goat anti-rabbit (Invitrogen A32732), Alexa Fluor 647 goat anti-mouse (Thermo Fisher A32733), and goat FITC-conjugated anti-GFP (Rockland Immunochemicals, 600-102-215) were used at 1:1,500 and incubated with samples at room temperature for 2 to 3 h. All larval fillet preparations were washed in PBT as described above and mounted in Vectashield (H-1000; Vector Laboratories, Burlingame, CA) or Vectashield Hardset (H-1400) prior to imaging.

To reduce background fluorescence and amplify signal, modifications to the staining protocol were made for the Cac immunohistochemistry experiments. Larval fillet preparations were incubated in rabbit anti-Cac as described above, followed by a 9-h wash in PBT at 4ºC. Samples were then incubated with 1:750 goat, anti-rabbit Biotin F(ab’)2 (Jackson 111-066-144) along with 1:150 mouse anti-Brp as described for the unc-13A immunohistochemistry experiments. Fillet preparations were then washed in PBT as described above, followed by two 1-h washes in PBT. Samples were blocked and stained with 1:750 Streptavidin-555+ (Thermo Fisher S32355) and 1:1,500 donkey anti-mouse 647 (Jackson 715-605-151).

### Airyscan Imaging.

As described recently (71), confocal and Airyscan imaging were performed on either a Zeiss LSM 880 or Zeiss LSM 980 microscope. Mounted fillet preparations were imaged with a ×63 oil immersion objective (NA 1.4, DIC; Zeiss) using Zen software (Zeiss Zen Black 2.3 SP1). Airyscan processing of imaging planes and channels was also performed in Zen.

Brp puncta surfaces and volumes were calculated using a custom Imaris (Oxford Instruments) routine. We calculated a surface from the SynapGCaMP signal for each NMJ to use as a mask when identifying Brp puncta. Brp puncta were then segmented using the Surfaces function, smoothed with a Gaussian kernel of 0.1 μm, and background subtracted with a 0.15 μm Gaussian kernel within the SynapGCaMP mask. Brp puncta in close proximity to each other were isolated with the Split Touching Objects function with a threshold of 0.15 μm. Low quality ROIs were removed with the Filter Seed Points function (Quality threshold: 59) and applying a minimum volume size (Number of Voxels: 10). To quantify the expression of Unc-13-A and Cac at the AZ, we summed the voxel intensities for these channels within each Brp punctum with the Imaris Spots function. Empirical cumulative distributions of Unc-13-A and Cac intensities were calculated for all Brp puncta for controls and RNAi knockdowns in R (ver 4.1.0).

## Supplementary Material

Appendix 01 (PDF)

## Data Availability

Code used for all RNA-seq data analysis steps will be available in a GitHub repository at https://github.com/isacofflab/ccyp_OK6_RNAi_2023 (91). QuaSOR custom code is available in the GitHub repository at https://github.com/newmanza/Newman_QuaSOR_2021; https://doi.org/10.5281/zenodo.5711302 (92). The RNA-seq libraries discussed in this publication and relevant metadata have been deposited in NCBI’s Gene Expression Omnibus (93) and are accessible through GEO Series GSE250462 and GSE301255 (94, 95). All other data are included in the manuscript and/or *SI Appendix*.
