# Supplementary material for: Deficiency in transmitter release triggers homeostatic transcriptional changes that increase presynaptic excitability
Source: Proc Natl Acad Sci U S A. 2025 Jul 29;122(31):e2322714122. doi: 10.1073/pnas.2322714122 (PMC12337328; doi:10.1073/pnas.2322714122)
Supplement: Supplementary file 1 — Appendix 01 (PDF) [file pnas.2322714122.sapp.pdf]

Supplementary Material

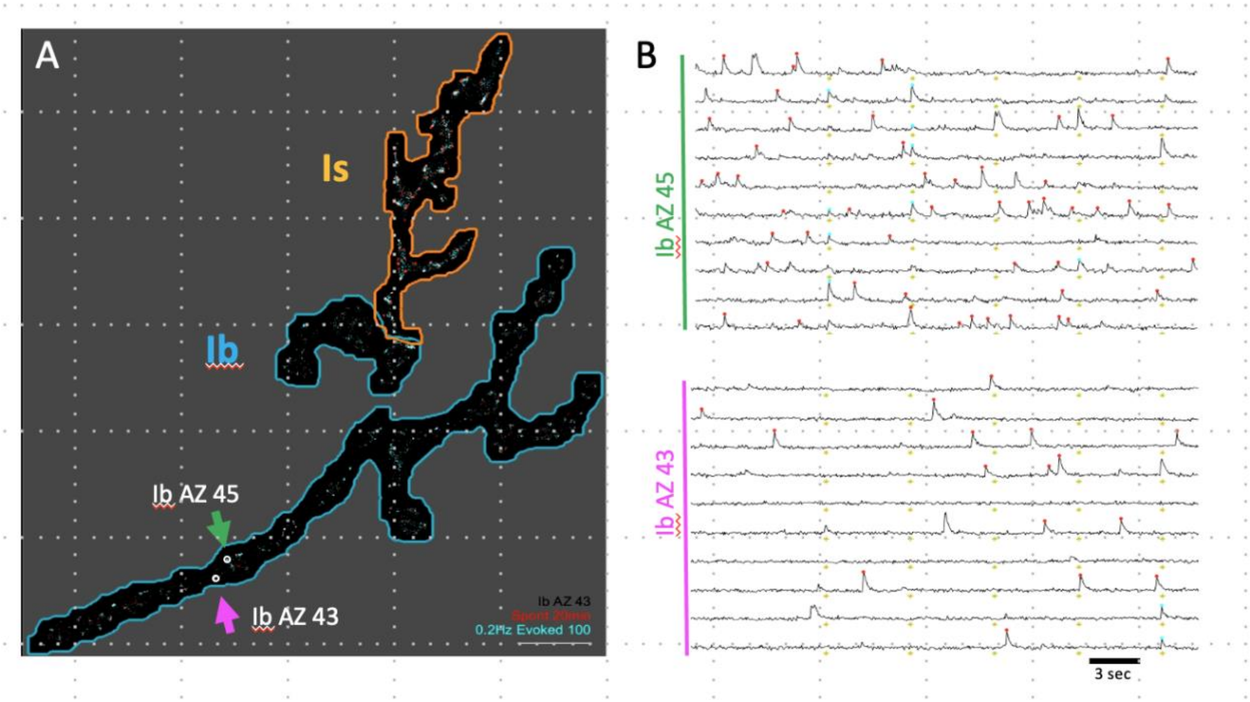

Figure S1) Quantal imaging of glutamatergic synaptic transmission by Ib motor neurons detected by SynapGCaMP6f in the *Drosophila* larval NMJ.

A, B) Abdominal segment showing spontaneous transmission (red) and transmission evoked by action potentials stimulated at 0.2 Hz (blue) by Ib MN4 and Is MSNISN to muscle 4.

A) Image of cumulative transmission.

B) Raster plots of transmission events at two identified synapses during first 300 sec period of a movie.

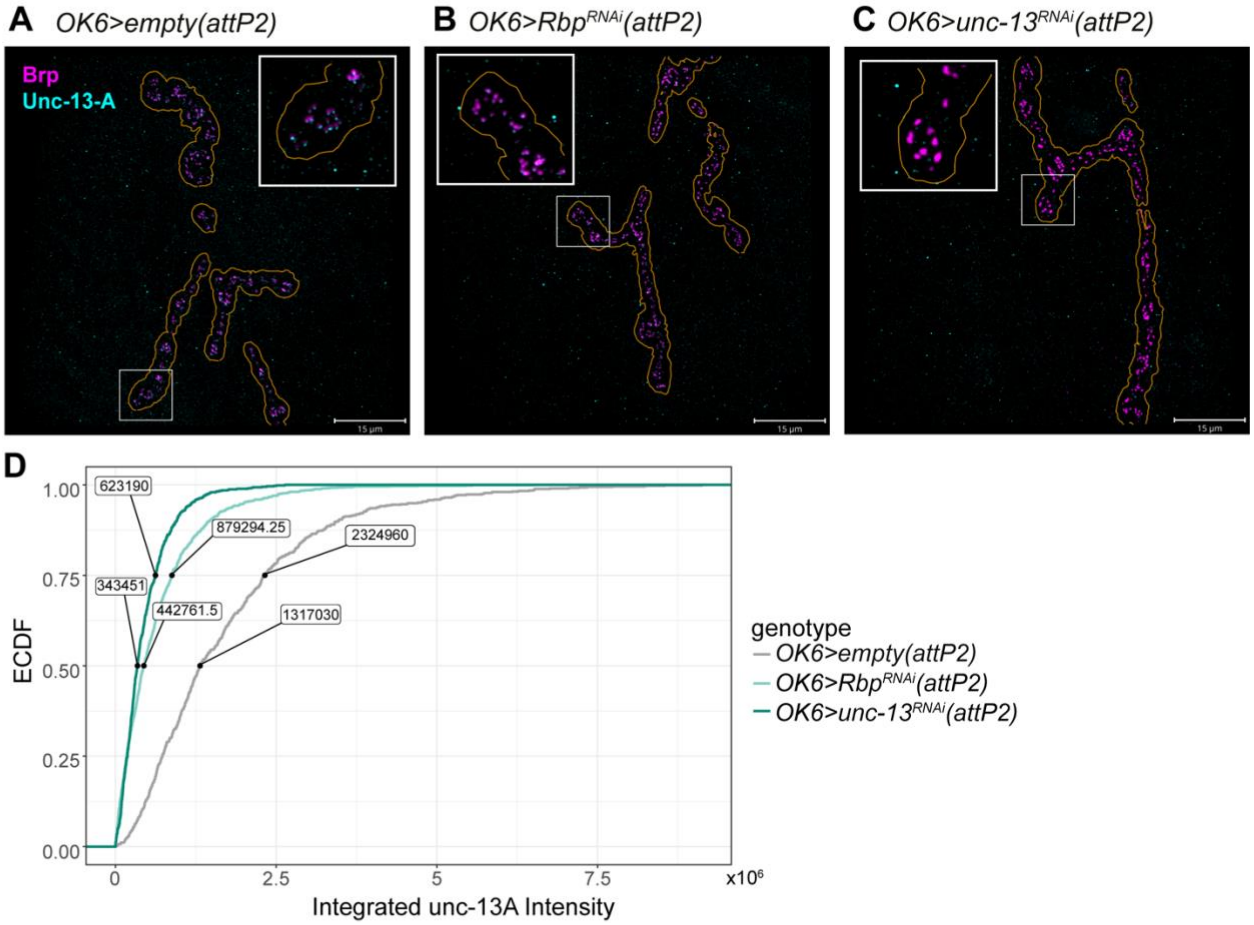

Figure S2) Rbp and Unc-13 knockdowns decrease Unc-13A at type Ib MN AZs.

A-C) Immunofluorescence stains of Brp (magenta) and Unc-13A (cyan) in type Ib MN terminals (orange outline) of control **(A)** and RNAi knockdowns, *OK6>Rbp*^{RNAi} **(B)** and *OK6>unc-13*^{RNAi} **(C)**. Scale bar: 15 µm. **D)** Cumulative distribution of summed Unc-13-A voxel intensities in controls and RNAi knockdowns (two-sided KS test, control v *Rbp*^{RNAi} *p*-value < 2.2e-16, control vs *unc-13*^{RNAi} *p*-value 2.2e-16). Values displayed are the 50th and 75th percentiles for each genotype. (Controls: n = 4 larvae, $n_{Brp\ puncta}$ = 711; *OK6> Rbp*^{RNAi}: n = 4 larvae; $n_{Brp\ puncta}$ = 1130; *OK6>unc-13*^{RNAi}: n = 4 larvae, $n_{Brp\ puncta}$ = 855).

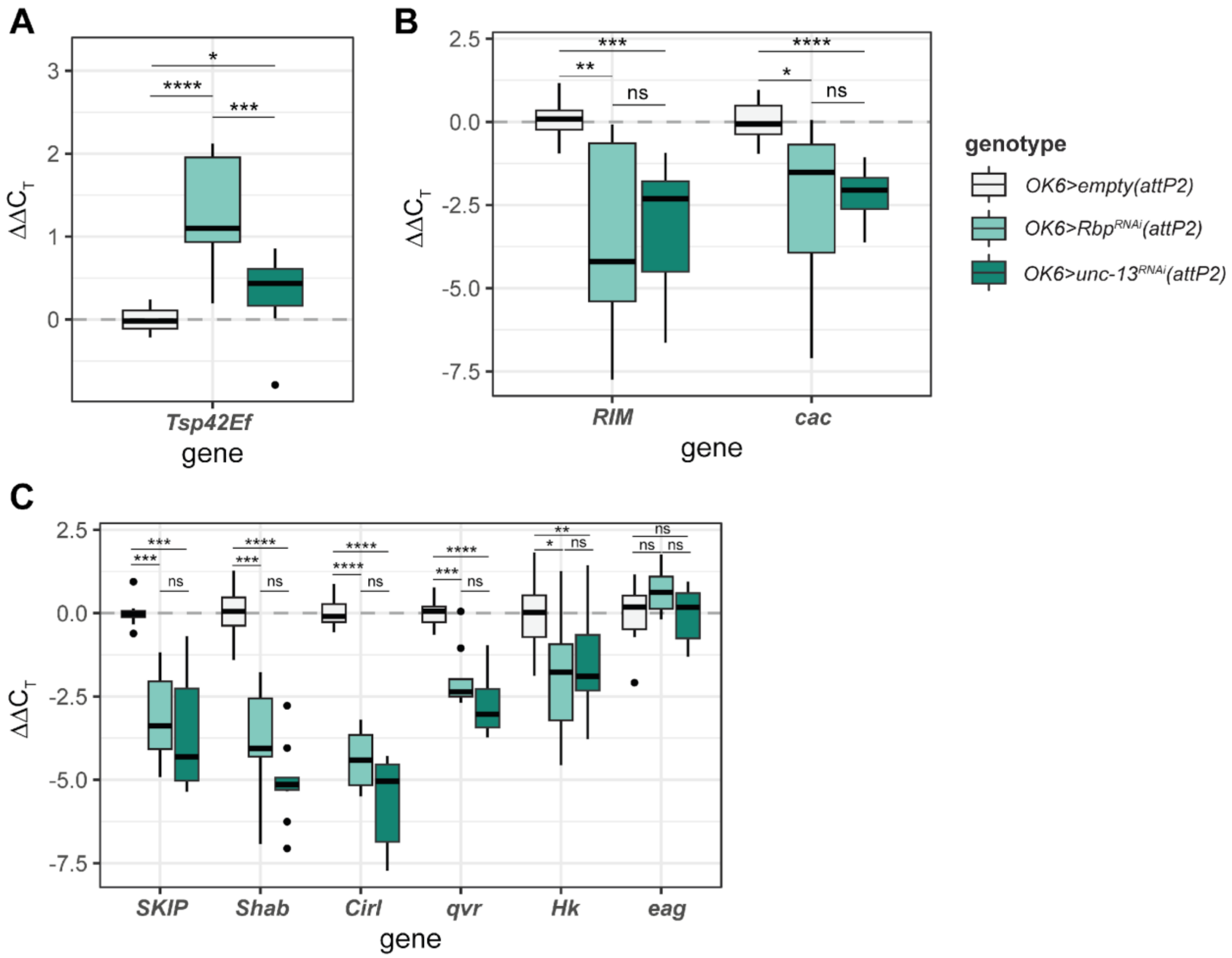

Figure S3) RT-PCR confirmation of changes in expression of AZ components and Kv channels in RNAi knockdowns.

A-C) $\Delta\Delta C_T$ values of *Tsp42Ef* (**A**), RSSPs (**B**), and Kv channels and their accessory proteins (**C**) in *OK6>Rbp*^{RNAi} and *OK6>unc-13*^{RNAi} (two-tailed T-test *p*-values, ns $p > 0.05$, * $p < 0.01$, ** $p < 0.001$, *** $p < 0.0001$, **** $p <$ 1e-05).

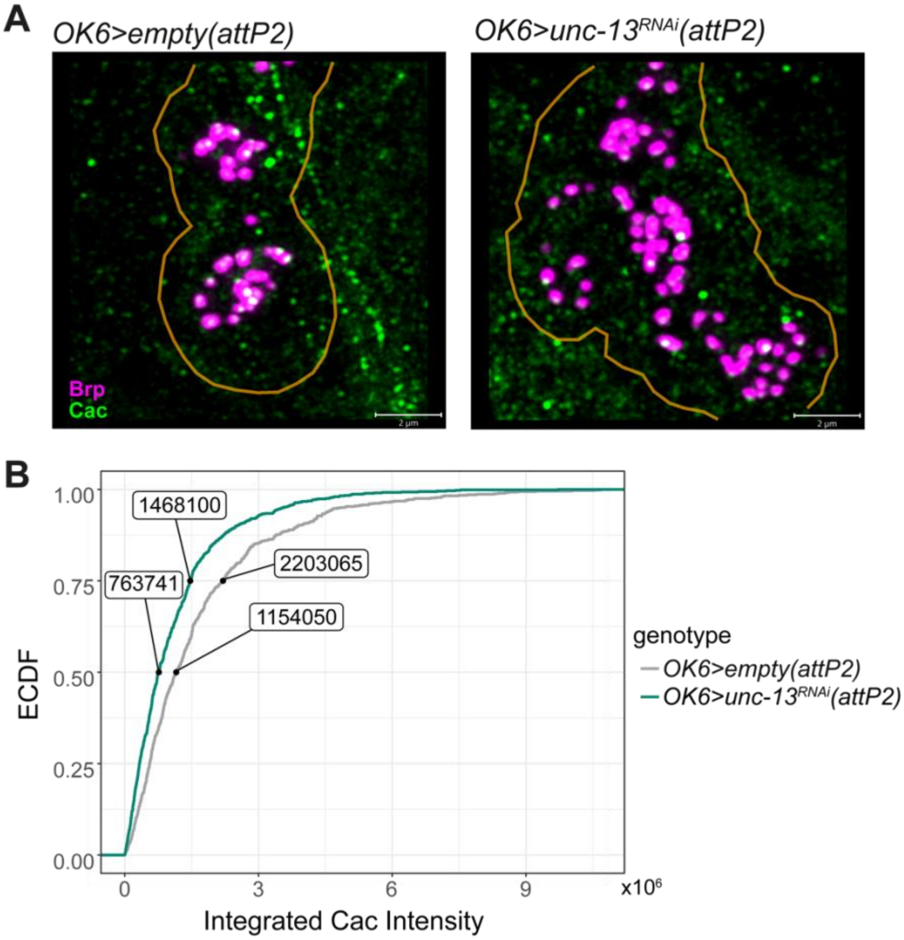

Figure S4) Unc-13 knockdown decreases Cac at type Ib MN AZs.

A) Immunofluorescence stains of Brp (magenta) and Cac (green) at type Ib MN terminals (orange outline) in controls **(left)** and *OK6>unc-13*^{RNAi} **(right)**. Scale bar: 2 µm. **B**) Empirical cumulative distribution of Cac fluorescence within Brp puncta between controls and *OK6>unc-13*^{RNAi} (two-sided KS test, p-value = 3.75e-09). Values shown are the 50th and 75th percentiles. (Controls: n = 8 larvae, $n_{\text{Brp puncta}}$ = 688; *OK6>unc-13*^{RNAi}: n = 8 larvae, $n_{\text{Brp puncta}}$ = 855).

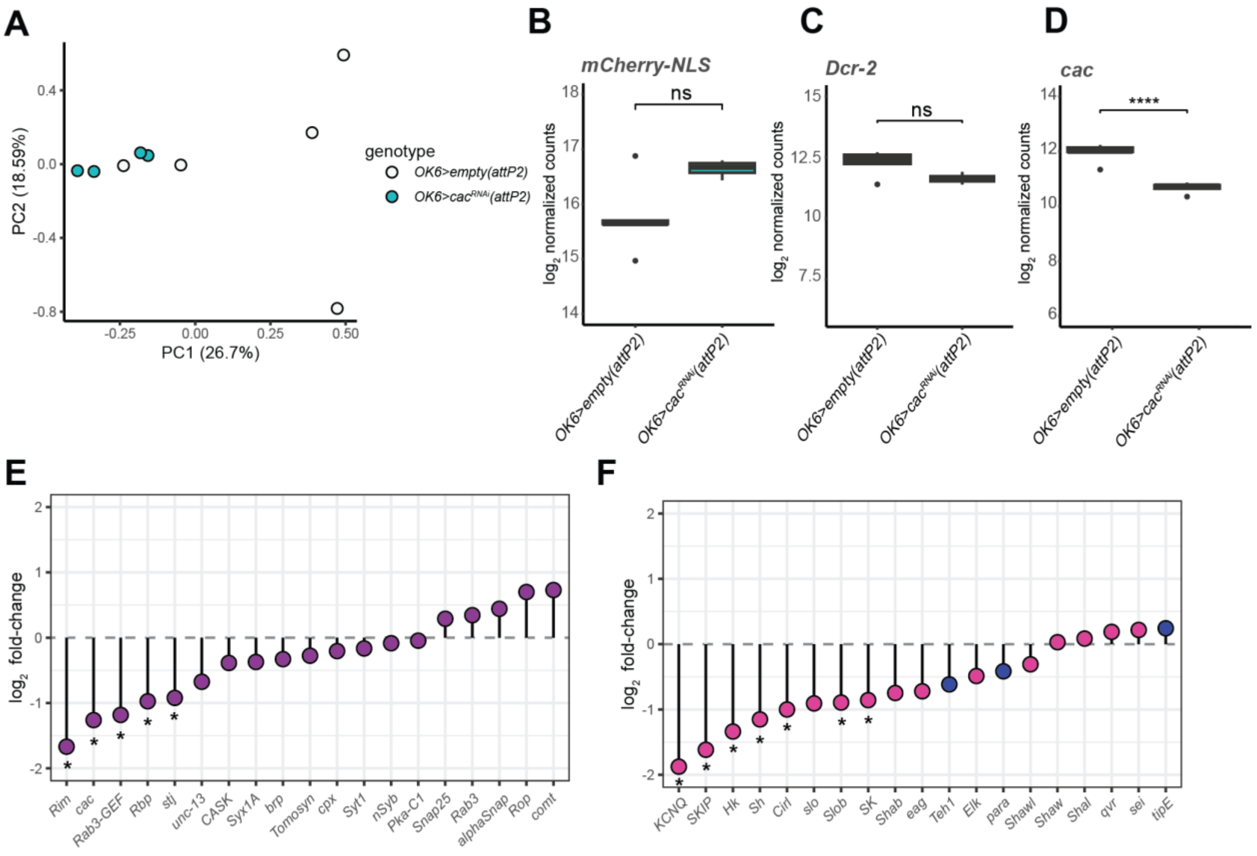

Figure S5) Knockdown of *cac* down-regulates K+ channels and their positive modulators without changing voltage-gated Na+ channel expression.

A) PCA plot of RNA-seq libraries from *OK6>empty(attP2)* control MNs (gray) and *OK6>cac^{RNAi}(attP2)* MNs (cyan). Axes are annotated by percent variance explained for each principal component. **B-D)** Boxplots of log2 normalized pseudocount expression of *mCherry-NLS***(B)**, *Dcr-2* **(C)** and *cac* **(D)** (BH-adjusted Wald test p-values, ns p > 0.05, * p < 0.01, ** p < 0.001, *** p < 0.0001, **** p < 1e-05). **E-F)** Differential expression of AZ components **(E)** and modulators of excitability **(F)**. Kv channels and Cirl are shown in magenta. Nav channels and auxiliary proteins are shown in blue. (BH adjusted *p*-value, * < 0.05).

Supplementary Table 1. Sample information for low-input RNA-seq of control and RNAi type I MNs.

Library	Run	Genotype	n_{VNCs}	n_{cells}	n_{genes}
OK6_ATTP2_001	1	*OK6>empty(attP2)*	12	6,414	8,899
OK6_ATTP2_002	1	*OK6>empty(attP2)*	12	6,414	8,941
OK6_ATTP2_003	1	*OK6>empty(attP2)*	12	6,414	8,835
OK6_UNC13_001	1	*OK6>unc-13^{RNAi}(attP2)*	19	1,753	8,178
OK6_UNC13_002	1	*OK6>unc-13^{RNAi}(attP2)*	19	1,753	8,304
OK6_UNC13_003	1	*OK6>unc-13^{RNAi}(attP2)*	19	1,753	8,213
OK6_ATTP2_101	2	*OK6>empty(attP2)*	10	3,530	8,662
OK6_ATTP2_102	2	*OK6>empty(attP2)*	10	3,530	8,495
OK6_ATTP2_103	2	*OK6>empty(attP2)*	10	3,530	8,583
OK6_RBP_101	2	*OK6>Rbp^{RNAi}(attP2)*	11	3,512	8,483
OK6_RBP_102	2	*OK6>Rbp^{RNAi}(attP2)*	11	3,512	8,667
OK6_RBP_103	2	*OK6>Rbp^{RNAi}(attP2)*	11	3,512	8,688

Run = Sort day; n_{VNCs} = number of VNCs sorted; n_{cells} = number of MNs sorted; n_{genes} = number of genes detected.

Supplementary Table 2. Top 20 up-regulated genes in *OK6>Rbp^{RNAi}* Type I MNs.

FlyBase ID	Gene symbol	log-fold-change	*p*-value	Adjusted *p*-value
FBgn0038343	Trissin	9.33	1.29E-12	1.77E-10
FBgn0011581	Ms	6.71	9.39E-50	8.92E-46
FBgn0000564	Eh	5.64	6.69E-05	1.05E-03
FBgn0050457	CG30457	5.24	1.68E-06	4.56E-05
FBgn0053527	SIFa	5.14	2.96E-05	5.29E-04
FBgn0000045	Act79B	5.04	1.46E-09	1.00E-07
FBgn0032096	Or30a	5.01	3.73E-03	2.58E-02
FBgn0000500	Dsk	4.44	2.00E-03	1.58E-02
FBgn0034709	Swim	4.35	4.67E-03	3.06E-02
FBgn0027109	NPF	4.05	4.81E-36	5.08E-33
FBgn0025878	wrapper	3.78	1.36E-03	1.17E-02
FBgn0032895	twit	3.06	2.57E-32	1.88E-29
FBgn0033135	Tsp42En	3.06	7.06E-06	1.60E-04
FBgn0000046	Act87E	3.01	7.17E-05	1.11E-03
FBgn0051370	CG31370	2.99	6.00E-03	3.71E-02
FBgn0032897	CG9336	2.88	3.31E-05	5.81E-04
FBgn0023534	CG17778	2.81	7.65E-12	8.66E-10
FBgn0001258	Ldh	2.80	2.79E-10	2.27E-08
FBgn0034709	Gsl1	2.69	1.29E-40	4.08E-37
FBgn0034583	CG10527	2.65	1.32E-32	1.09E-29

Supplementary Table 3. Top 20 up-regulated genes in *OK6>unc-13^{RNAi}* Type I MNs.

FlyBase ID	Gene symbol	log-fold-change	*p*-value	Adjusted *p*-value
FBgn0027109	NPF	7.81	1.04E-129	9.85E-126
FBgn0050457	CG30457	7.20	3.66E-11	9.38E-09
FBgn0038349	AOX3	7.13	1.16E-04	4.03E-03
FBgn0032096	Or30a	7.08	3.72E-05	1.58E-03
FBgn0000045	Act79B	6.69	9.91E-16	5.22E-13
FBgn0025878	wrapper	6.42	3.70E-08	4.88E-06
FBgn0036146	nkt	6.35	4.31E-05	1.79E-03
FBgn0039332	alrm	6.03	1.94E-17	1.15E-14
FBgn0028940	Cyp28a5	5.96	2.19E-03	3.53E-02
FBgn0041087	wun2	5.44	2.54E-06	1.61E-04
FBgn0038799	MFS9	5.29	2.00E-13	7.59E-11
FBgn0010389	htl	5.20	2.13E-03	3.47E-02
FBgn0030258	CG1552	5.13	1.36E-04	4.46E-03
FBgn0033268	Obp44a	5.09	5.99E-48	1.90E-44
FBgn0039915	Gat	5.07	5.01E-07	4.37E-05
FBgn0034588	CG9394	4.99	1.99E-03	3.29E-02
FBgn0012037	Ance	4.94	9.81E-04	1.93E-02
FBgn0262531	CG43085	4.78	1.16E-04	4.03E-03
FBgn0030304	Cyp4g15	4.67	5.02E-05	2.03E-03
FBgn0001145	Gs2	4.63	1.45E-17	9.17E-15

Supplementary Table 4. Expression of AZ component genes in *OK6>Rbp^{RNAi}* Type I MNs.

FlyBase ID	Gene symbol	log-fold-change	*p*-value	Adjusted *p*-value
FBgn0053547	Rim	-1.35	1.93E-13	3.40E-11
FBgn0025726	unc-13	-1.19	2.17E-10	1.79E-08
FBgn0004574	Rop	1.75	8.81E-09	5.00E-07
FBgn0350791	alphaSnap	1.54	2.85E-09	9.84E-06
FBgn0041605	cpx	1.13	6.00E-06	1.38E-04
FBgn0262483	Rbp	-1.71	1.25E-05	2.59E-04
FBgn0000346	comt	0.758	3.38E-04	3.96E-03
FBgn0261041	stj	-0.658	6.51E-04	6.72E-03
FBgn0263111	cac	-0.962	9.01E-04	8.60E-03
FBgn0000273	Pka-C1	0.893	1.81E-03	1.46E-02
FBgn0030613	Rab3-GEF	-0.703	7.54E-02	0.231
FBgn0259246	brp	0.433	0.108	0.291
FBgn0013759	CASK	-0.297	0.163	0.376
FBgn0030412	Tomosyn	-0.463	0.170	0.387
FBgn0013343	Syx1A	0.294	0.185	0.407
FBgn0005586	Rab3	0.347	0.368	0.600
FBgn0004242	Syt1	0.270	0.400	0.629
FBgn0013342	nSyb	0.148	0.568	0.764
FBgn0011288	Snap25	-0.093	0.792	0.901

Supplementary Table 5. Expression of AZ component genes in *OK6>unc-13^{RNAi}* Type I MNs.

FlyBase ID	Gene symbol	log-fold-change	*p*-value	Adjusted *p*-value
FBgn0025726	unc-13	-1.19	3.38E-10	7.13E-08
FBgn0053547	Rim	-1.11	1.47E-09	2.74E-07
FBgn0263111	cac	-1.39	1.89E-06	1.26E-04
FBgn0000346	comt	0.685	1.25E-03	2.37E-02
FBgn0041605	cpx	0.793	1.57E-03	2.75E-02
FBgn0261041	stj	-0.561	4.22E-03	5.53E-02
FBgn0004574	Rop	0.804	8.42E-03	8.79E-02
FBgn0013343	Syx1A	-0.551	1.30E-02	0.117
FBgn0030613	Rab3-GEF	-0.961	1.62E-02	0.133
FBgn0262483	Rbp	-0.722	6.50E-02	0.303
FBgn0013759	CASK	-0.360	9.20E-02	0.359
FBgn0259246	brp	-0.433	0.110	0.393
FBgn0004242	Syt1	-0.450	0.161	0.475
FBgn0030412	Tomosyn	-0.426	0.210	0.533
FBgn0011288	Snap25	-0.417	0.236	0.564
FBgn0250791	alphaSnap	0.256	0.392	0.704
FBgn0013342	nSyb	-0.110	0.672	0.875
FBgn0005586	Rab3	0.093	0.809	0.929
FBgn0000273	Pka-C1	0.043	0.882	0.958

Supplementary Table 6. Expression of K_v and Na_v genes in *OK6>Rbp^{RNAi}* Type I MNs.

**K+ channels and auxiliary subunits**				
FlyBase ID	Gene symbol	log-fold-change	*p*-value	Adjusted *p*-value
FBgn0051163	SKIP	-1.55	2.25E-15	5.08E-13
FBgn0033313	Cirl	-1.41	3.27E-12	3.84E-10
FBgn0260499	qvr	-0.766	4.88E-05	7.98E-04
FBgn0262593	Shab	-0.861	5.20E-05	8.39E-04
FBgn0011589	Elk	-1.10	1.20E-04	1.70E-03
FBgn0033494	KCNQ	-1.33	5.38E-04	5.81E-03
FBgn0261698	SLO2	-1.04	7.75E-04	7.62E-03
FBgn0264087	Slob	0.848	9.04E-04	8.62E-03
FBgn0003380	Sh	-0.964	1.06E-03	9.77E-03
FBgn0263220	Hk	-0.613	1.68E-03	1.39E-02
FBgn0038165	Task6	-0.847	3.26E-03	2.30E-02
FBgn0085395	Shawl	0.897	5.30E-03	3.38E-02
FBgn0003429	slo	-0.526	8.91E-03	4.99E-02
FBgn0005564	Shal	-0.349	7.60E-02	0.231
FBgn0003386	Shaw	-0.437	0.113	0.299
FBgn0029761	SK	-0.607	0.147	0.352
FBgn0037690	Task7	0.391	0.155	0.364
FBgn0003353	sei	-0.341	0.341	0.575
FBgn0000535	eag	-0.253	0.459	0.679
**Voltage-gated Na+ channels and auxiliary subunits**				
FlyBase ID	Gene symbol	log-fold-change	*p*-value	Adjusted *p*-value
FBgn0285944	para	-0.657	0.055	0.629
FBgn0037766	Teh1	-0.482	0.121	0.764
FBgn0003710	tipE	0.007	0.969	0.901

Supplementary Table 7. Expression of K_v and Na_v genes in *OK6>unc-13^{RNAi}* Type I MNs.

**K+ channels and auxiliary subunits**				
FlyBase ID	Gene symbol	log-fold-change	*p*-value	Adjusted *p*-value
FBgn0033313	Cirl	-1.51	3.06E-13	1.08E-10
FBgn0051163	SKIP	-1.21	1.45E-09	2.74E-07
FBgn0263220	Hk	-0.886	8.06E-06	4.28E-04
FBgn0003380	Sh	-01.24	2.75E-05	1.23E-03
FBgn0260499	qvr	-0.705	2.19E-04	6.37E-03
FBgn0262593	Shab	-0.766	3.70E-04	9.38E-03
FBgn0011589	Elk	-0.975	9.77E-04	1.92E-02
FBgn0262593	SLO2	-0.866	5.70E-03	6.75E-02
FBgn0003429	slo	-0.540	7.92E-03	8.48E-02
FBgn0000535	eag	-0.855	1.38E-02	0.120
FBgn0033494	KCNQ	-0.947	1.71E-02	0.138
FBgn0085395	Shawl	-0.723	2.76E-02	0.184
FBgn0264087	Slob	0.523	4.25E-02	0.238
FBgn0038165	Task6	-0.569	4.99E-02	0.259
FBgn0029761	SK	-0.607	0.064	0.299
FBgn0003386	Shaw	-0.429	0.068	0.311
FBgn0005564	Shal	-0.166	0.406	0.717
FBgn0037690	Task7	0.164	0.562	0.817
FBgn0003353	sei	0.104	0.775	0.914
**Voltage-gated Na+ channels and auxiliary subunits**				
FlyBase ID	Gene symbol	log-fold-change	*p*-value	Adjusted *p*-value
FBgn0285944	para	-0.773	2.40E-02	0.169
FBgn0037766	Teh1	-0.429	0.174	0.491
FBgn0003710	tipE	-0.166	0.380	0.695

Supplementary Table 8. Expression of neuropeptide receptor genes in *OK6>Rbp^{RNAi}* Type I MNs.

FlyBase ID	Gene symbol	log-fold-change	*p*-value	Adjusted *p*-value
FBgn0037408	NPFR	-1.11	1.77E-04	3.62E-03
FBgn0004841	TkR86C	0.078	0.859	0.939
FBgn0029723	Proc-R	-3.71	0.088	0.253
FBgn0038139	PK2-R2	-0.510	0.287	0.561
FBgn0038140	PK2-R1	-1.03	6.75E-04	9.95E-03
FBgn0038201	PK1-R	-0.852	0.205	0.470
FBgn0038874	ETHR	0.143	0.733	0.879
FBgn0038880	SIFaR	-0.154	0.766	0.895
FBgn0039396	CCAP-R	-1.58	1.07E-05	4.15E-04
FBgn0004842	RYa-R	-0.573	0.291	0.566
FBgn0039595	AstA-R2	-1.76	1.02E-06	5.97E-05
FBgn0004622	TkR99D	0.184	0.490	0.732
FBgn0264002	MsR2	-0.833	0.119	0.343
FBgn0035331	MsR1	-0.832	0.060	0.226
FBgn0035385	FMRFaR	-0.233	0.614	0.815
FBgn0035610	Lkr	-0.914	0.009	0.061
FBgn0053696	CNMaR	-0.794	0.113	0.331
FBgn0036278	CrzR	-0.908	0.037	0.165
FBgn0036789	AstC-R2	-1.86	2.35E-07	1.85E-05
FBgn0036790	AstC-R1	-1.92	0.091	0.288
FBgn0036934	sNPF-R	-1.20	8.29E-04	0.012
FBgn0037100	CapaR	-0.548	0.553	0.776
FBgn0033058	CCHa2-R	-0.067	0.784	0.904
FBgn0033744	Dh44-R2	-0.071	0.860	0.939

Supplementary Table 9. Expression of neuropeptide receptor genes in *OK6>unc-13*^{RNAi} Type I MNs.

FlyBase ID	Gene symbol	log-fold-change	*p*-value	Adjusted *p*-value
FBgn0037408	NPFR	-1.0766581	3.49E-04	0.014
FBgn0004841	TkR86C	0.57818307	0.192	0.587
FBgn0029723	Proc-R	1.46	0.443	0.745
FBgn0038139	PK2-R2	-0.5383246	0.270	0.667
FBgn0038140	PK2-R1	-0.8116457	9.61E-03	0.128
FBgn0038201	PK1-R	-0.4089712	0.551	0.860
FBgn0038874	ETHR	0.71183909	0.093	0.436
FBgn0038880	SIFaR	-3.2700512	7.68E-08	1.42E-05
FBgn0039396	CCAP-R	-1.1086678	2.97E-03	0.062
FBgn0004842	RYa-R	0.03736253	0.946	0.987
FBgn0039595	AstA-R2	-0.8153448	0.025	0.221
FBgn0004622	TkR99D	0.75852444	4.84E-03	0.084
FBgn0264002	MsR2	-0.2289584	0.674	0.911
FBgn0035331	MsR1	-0.2848718	0.523	0.845
FBgn0035385	FMRFaR	0.05374804	0.909	0.977
FBgn0035610	Lkr	-0.4722641	0.183	0.576
FBgn0053696	CNMaR	0.31123026	0.536	0.853
FBgn0036278	CrzR	0.10520534	0.810	0.949
FBgn0036789	AstC-R2	-1.418269	1.00E-04	5.36E-03
FBgn0036790	AstC-R1	-0.839652	0.466	0.814
FBgn0036934	sNPF-R	-0.4859298	0.178	0.571
FBgn0037100	CapaR	0.91923605	0.320	0.707
FBgn0033058	CCHa2-R	0.03495696	0.888	0.973
FBgn0033744	Dh44-R2	-0.6429156	0.144	0.524

FBgn0052843	Dh31-R	0.09442688	0.797	0.945
FBgn0033932	Dh44-R1	-0.3911786	0.348	0.729
FBgn0050106	CCHa1-R	-0.8504323	0.124	0.493
FBgn0266429	AstA-R1	-1.1907176	0.059	0.351
FBgn0029768	SPR	-1.5134016	5.36E-08	1.04E-05
FBgn0259231	CCKLR-17D1	0.13379524	0.732	0.930
FBgn0030954	CCKLR-17D3	-0.0631631	0.902	0.977
FBgn0085410	TrissinR	-0.6916678	0.021	0.201
FBgn0003255	rk	-0.9592637	0.150	0.534

Supplementary Table 10. Expression of ionotropic receptor genes in *OK6>Rbp^{RNAi}* Type I MNs.

**Cholinergic Receptors**				
FlyBase ID	Gene symbol	log-fold-change	*p*-value	Adjusted *p*-value
FBgn0032151	nAChRalpha6	-1.27	4.54E-08	2.00E-06
FBgn0000036	nAChRalpha1	-1.25	4.94E-04	5.46E-03
FBgn0028875	nAChRalpha5	-0.997	4.52E-04	5.06E-03
FBgn0015519	nAChRalpha3	-0.369	0.402	0.630
FBgn0266347	nAChRalpha4	-0.298	0.215	0.447
FBgn0086778	nAChRalpha7	0.014	0.945	0.977
FBgn0004118	nAChRbeta2	0.484	8.17E-02	0.242
FBgn0000039	nAChRalpha2	0.530	7.37E-03	4.31E-02
FBgn0000038	nAChRbeta1	0.546	3.92E-02	0.147
**GABAergic Receptors**				
FlyBase ID	Gene symbol	log-fold-change	*p*-value	Adjusted *p*-value
FBgn0001134	Grd	-3.02	0.131	0.330
FBgn0033558	CG12344	-1.80	9.38E-02	0.265
FBgn0030707	CG8916	-0.814	0.605	0.788
FBgn0004244	Rdl	-0.721	9.13E-04	8.70E-03
FBgn0010240	Lcch3	0.135	0.632	0.806
**Glutamatergic Receptors**				
FlyBase ID	Gene symbol	log-fold-change	*p*-value	Adjusted *p*-value
FBgn0039916	Ekar	-3.81	4.17E-04	4.74E-03
FBgn0051201	GluRIIE	-1.75	5.38E-02	0.183
FBgn0264000	GluRIB	-0.94	1.63E-05	3.23E-04
FBgn0024963	GluCalpha	-0.63	2.69E-03	1.99E-02

FBgn0004619	GluRIA	-0.33	0.201	0.428
FBgn0039927	CG11155	-0.27	0.168	0.383
FBgn0053513	Nmdar2	-0.188	0.371	0.602
FBgn0010399	Nmdar1	0.066	0.721	0.860
FBgn0038837	KaiR1D	0.26	0.290	0.528
FBgn0038840	Grik	0.55	0.634	0.808

Supplementary Table 11. Expression of ionotropic receptor genes in *OK6>unc-13^{RNAi}* Type I MNs.

**Cholinergic Receptors**				
FlyBase ID	Gene symbol	log-fold-change	*p*-value	Adjusted *p*-value
FBgn0032151	nAChRalpha6	-1.34	1.15E-08	1.80E-06
FBgn0015519	nAChRalpha3	-1.02	2.22E-02	0.162
FBgn0000036	nAChRalpha1	-0.789	2.84E-02	0.188
FBgn0028875	nAChRalpha5	-0.648	0.234	0.167
FBgn0086778	nAChRalpha7	-0.428	4.21E-02	0.237
FBgn0266347	nAChRalpha4	-0.199	0.416	0.725
FBgn0000039	nAChRalpha2	-0.170	3.96E-01	0.708
FBgn0004118	nAChRbeta2	0.046	0.870	0.953
FBgn0000038	nAChRbeta1	0.118	0.657	0.868
**GABAergic Receptors**				
FlyBase ID	Gene symbol	log-fold-change	*p*-value	Adjusted *p*-value
FBgn0001134	Grd	-4.25	5.31E-02	0.271
FBgn0004244	Rdl	-1.13	1.93E-07	1.95E-05
FBgn0010240	Lcch3	0.310	0.278	0.607
FBgn0030707	CG8916	0.214	0.894	0.964
FBgn0033558	CG12344	0.223	0.833	0.939
**Glutamatergic Receptors**				
FlyBase ID	Gene symbol	log-fold-change	*p*-value	Adjusted *p*-value
FBgn0039916	Ekar	-1.78	8.60E-02	0.348
FBgn0024963	GluCalpha	-1.01	1.91E-06	1.26E-04
FBgn0264000	GluRIB	-0.87	1.01E-04	3.60E-03
FBgn0039927	CG11155	-0.52	8.76E-03	8.99E-02

FBgn0004619	GluRIA	-0.43	0.105	0.384
FBgn0053513	Nmdar2	-0.29	0.168	0.483
FBgn0051201	GluRIIE	-0.21	0.814	0.930
FBgn0038837	KaiR1D	0.01	0.974	0.991
FBgn0010399	Nmdar1	0.49	0.957	9.57E-02
FBgn0038840	Grik	0.61	0.608	0.843

Supplementary Table 12. Sample information for low-input RNA-seq for experiments in Figure 5.

Library	Run	Genotype	n_{VNCs}	n_{cells}	n_{genes}
attP2_2	1	*OK6>empty(attP2)*	17	8234	10,408
attP2_4	2	*OK6>empty(attP2)*	14	8199	10,919
attP2_5	3	*OK6>empty(attP2)*	17	9000	10,796
attP2_6	4	*OK6>empty(attP2)*	14	4186	10,142
attP2_8	5	*OK6>empty(attP2)*	24	7422	10,558
CacRNAi_6	6	*OK6>cac^{RNAi}(attP2)*	19	8574	10,426
CacRNAi_7	7	*OK6>cac^{RNAi}(attP2)*	19	4862	10,112
CacRNAi_8	7	*OK6>cac^{RNAi}(attP2)*	18	6166	10,563
CacRNAi_9	8	*OK6>cac^{RNAi}(attP2)*	19	7878	10,718

Run = Sort day; n_{VNCs} = number of VNCs sorted; n_{cells} = number of MNs sorted; n_{genes} = number of genes detected.

Supplementary Table 13. Top 20 up-regulated genes in *OK6>cac^{RNAi}* Type I MNs.

FlyBase ID	Gene symbol	log-fold-change	*p*-value	Adjusted *p*-value
FBgn0039722	Capa	4.32	3.66E-04	0.024
FBgn0011581	lncRNA:CR45128	3.25	3.29E-04	0.024
FBgn0051159	mRRF2	2.49	7.27E-04	0.036
FBgn0267511	28sRNA-Psi:CR45851	2.21	2.73E-07	2.43E-04
FBgn0039003	wfs1	1.73	6.78E-04	0.035
FBgn0001186	Hex-A	1.72	3.91E-05	0.006
FBgn0032153	CG4537	1.72	6.29E-04	0.034
FBgn0034245	UQCR-6.4	1.69	3.58E-04	0.025
FBgn0033085	CG15908	1.41	1.14E-03	0.048
FBgn0061198	HSPC300	1.36	7.42E-04	0.03**7**
FBgn0030292	CG11752	1.31	1.05E-03	0.046
FBgn0086558	Ubi-p5E	1.31	5.05E-05	0.007
FBgn0003071	Pfk	1.26	6.25E-04	0.034
FBgn0027794	Lrpprc2	1.24	4.06E-04	0.026
FBgn0040079	pkaap	1.23	7.87E-04	0.038
FBgn0058002	ND-AGGG	1.21	1.19E-03	0.049
FBgn0031266	Sf3b1	1.19	2.48E-04	0.020
FBgn0003943	Ubi-p63E	1.13	2.00E-05	0.004
FBgn0036342	CG11279	1.13	1.08E-03	0.046
FBgn0064225	RpL5	1.04	5.91E-04	0.033

Supplementary Table 14. Expression of ionotropic receptor genes in *OK6>cac^{RNAi}* Type I MNs.

**Cholinergic Receptors**				
FlyBase ID	Gene symbol	log-fold-change	*p*-value	Adjusted *p*-value
FBgn0000038	nAChRbeta	-1.47	2.06E-06	8.51E-04
FBgn0032151	nAChRalpha6	-1.09	8.40E-05	0.010
FBgn0000036	nAChRalpha1	-1.00	2.17E-04	0.018
FBgn0015519	nAChRalpha3	-0.905	6.29E-04	0.034
FBgn0086778	nAChRalpha7	-0.904	1.04E-04	0.011
FBgn0028875	nAChRalpha5	-0.866	5.92E-04	0.033
FBgn0266347	nAChRalpha4	-0.328	0.153	0.485
FBgn0004118	nAChRbeta2	-0.245	0.301	0.638
FBgn0000039	nAChRalpha2	0.031	0.864	0.954
**GABAergic Receptors**				
FlyBase ID	Gene symbol	log-fold-change	*p*-value	Adjusted *p*-value
FBgn0004244	Rdl	-1.0280187	3.49E-04	0.024
FBgn0033558	CG12344	-0.8990250	0.220	0.564
FBgn0001134	Grd	-0.4593383	0.568	0.819
FBgn0010240	Lcch3	-0.2288639	0.270	0.616
FBgn0030707	CG8916	-0.2239424	0.622	0.848
**Glutamatergic Receptors**				
FlyBase ID	Gene symbol	log-fold-change	*p*-value	Adjusted *p*-value
FBgn0039916	Ekar	-1.62787152	0.018	0.190
FBgn0038840	Grik	-0.72852798	0.157	0.488
FBgn0024963	GluClalpha	-0.32662292	0.092	0.394

FBgn0039927	CG11155	-0.30030452	0.124	0.443
FBgn0010399	Nmdar1	-0.11981967	0.618	0.846
FBgn0053513	Nmdar2	0.02944238	0.899	0.966
FBgn0264000	GluRIB	0.17940426	0.535	0.800
FBgn0038837	KaiR1D	0.28657337	0.244	0.586
FBgn0004619	GluRIA	0.40755762	0.030	0.240
FBgn0051201	GluRIIE	0.44174132	0.335	0.666
